# Ten years of demographic modelling of divergence and speciation in the sea

**DOI:** 10.1111/eva.13428

**Published:** 2022-06-26

**Authors:** Aurélien De Jode, Alan Le Moan, Kerstin Johannesson, Rui Faria, Sean Stankowski, Anja Marie Westram, Roger K. Butlin, Marina Rafajlović, Christelle Fraïsse

**Affiliations:** ^1^ Department of Marine Sciences‐Tjärnö University of Gothenburg Gothenburg Sweden; ^2^ CIBIO, Centro de Investigação em Biodiversidade e Recursos Genéticos, InBIO Laboratório Associado Universidade do Porto Vairão Portugal; ^3^ BIOPOLIS Program in Genomics, Biodiversity and Land Planning CIBIO Vairão Portugal; ^4^ Institute of Science and Technology Austria (IST Austria) Klosterneuburg Austria; ^5^ Faculty of Biosciences and Aquaculture Nord University Bodø Norway; ^6^ Ecology and Evolutionary Biology, School of Biosciences The University of Sheffield Sheffield UK; ^7^ Department of Marine Sciences University of Gothenburg Gothenburg Sweden; ^8^ UMR 8198 – Evo‐Eco‐Paleo, CNRS Univ. Lille Lille France

**Keywords:** demographic inference, grey zone of speciation, heterogeneous gene flow, marine speciation, primary and secondary contact, reproductive isolation

## Abstract

Understanding population divergence that eventually leads to speciation is essential for evolutionary biology. High species diversity in the sea was regarded as a paradox when strict allopatry was considered necessary for most speciation events because geographical barriers seemed largely absent in the sea, and many marine species have high dispersal capacities. Combining genome‐wide data with demographic modelling to infer the demographic history of divergence has introduced new ways to address this classical issue. These models assume an ancestral population that splits into two subpopulations diverging according to different scenarios that allow tests for periods of gene flow. Models can also test for heterogeneities in population sizes and migration rates along the genome to account, respectively, for background selection and selection against introgressed ancestry. To investigate how barriers to gene flow arise in the sea, we compiled studies modelling the demographic history of divergence in marine organisms and extracted preferred demographic scenarios together with estimates of demographic parameters. These studies show that geographical barriers to gene flow do exist in the sea but that divergence can also occur without strict isolation. Heterogeneity of gene flow was detected in most population pairs suggesting the predominance of semipermeable barriers during divergence. We found a weak positive relationship between the fraction of the genome experiencing reduced gene flow and levels of genome‐wide differentiation. Furthermore, we found that the upper bound of the ‘grey zone of speciation’ for our dataset extended beyond that found before, implying that gene flow between diverging taxa is possible at higher levels of divergence than previously thought. Finally, we list recommendations for further strengthening the use of demographic modelling in speciation research. These include a more balanced representation of taxa, more consistent and comprehensive modelling, clear reporting of results and simulation studies to rule out nonbiological explanations for general results.

## 1 INTRODUCTION

### 1.1 Divergence and speciation in the sea

Understanding the processes that drive divergence and speciation is a central goal in evolutionary biology (Butlin et al., 2012; Coyne & Orr, 2004; Dobzhansky, 1952; Mayr, 1947; Nosil, 2012). One of the most intense debates among speciation researchers has been about the geographical context in which reproductive isolation evolves, traditionally classified as allopatric, parapatric and sympatric speciation (Coyne & Orr, 2004). For a long time, allopatric speciation was considered the predominant mode (Coyne & Orr, 2004; Mayr, 1963). However, this idea was challenged, not least by marine taxa: because geographical barriers (Box 1) in the sea seemed rare and many marine organisms have high dispersal capabilities, it was difficult to explain the enormous marine biodiversity with an allopatric model. A potential solution to this ‘marine speciation paradox’ (Bierne et al., 2003; Palumbi, 1994) arose when empirical studies (on both terrestrial and marine taxa), in combination with theoretical work, showed that speciation can and does occur without complete geographical isolation (Abbott et al., 2013; Coyne & Orr, 2004; Nosil, 2012).

BOX 1 1 *Geographical or physical barriers to gene flow*. In this review, we used the term geographical barriers to refer to both geographical barriers such as distance between populations and physical barriers such as oceanographic currents.

*Demographic methods used*. Despite the various inferential methods developed for demographic inferences, only three were used in the studies we examined.

- **δaδi:** a likelihood‐based inference software using a diffusion approximation on the site‐frequency spectrum (Gutenkunst et al., 2010).
- ***moments*:** a likelihood‐based inference software using ordinary differential equations on the site‐frequency spectrum (Jouganous et al., 2017).
- **ABC:** Approximate Bayesian Computation, a simulation‐based inference method using an array of summary statistics (e.g. DILS software, Fraïsse et al., 2021a).

*Demographic models tested*. Five scenarios with different temporal patterns of gene flow were tested in the studies we examined: Strict Isolation, Isolation with Migration, Ancient Migration, SC and Periodic Connectivity (see cartoon below). For those scenarios of divergence with gene flow (i.e. all except for SI), a model of heterogeneity in effective migration rates across the genome (hetM) was systematically tested in our dataset. Genome‐wide heterogeneity of effective population size (hetN) can be expected in all scenarios, but it was tested in only a fraction of the studies we examined.
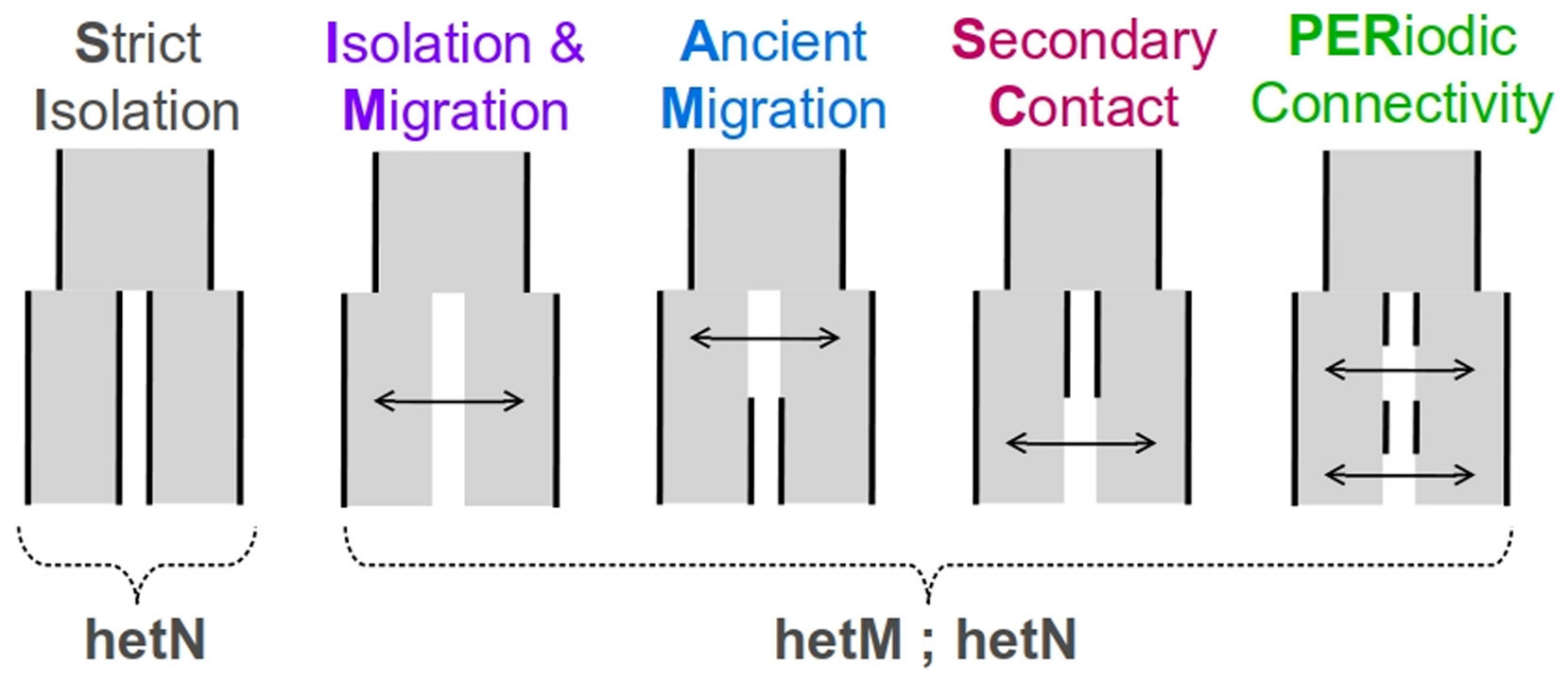

- **SI, Strict Isolation**: divergence between the two lineages occurs in the absence of gene flow.
- **IM, Isolation with migration**: divergence with continuous gene flow between the two lineages (arrow).
- **AM, Ancient Migration**: divergence starts in the presence of gene flow (arrow), followed by a single period of isolation.
- **SC, Secondary contact**: divergence starts in isolation, followed by a single period of gene flow (arrow).
- **PER, Periodic connectivity**: divergence with alternating phases of isolation and contacts (multiple periods).
- **hetM:** a demographic model with heterogeneous effective gene flow across the genome. Heterogeneity was modelled in two possible ways: (i) the effective migration rate is beta distributed across the genome (Roux et al., 2013), (ii) two categories of loci have different migration rates (Tine et al., 2014), with loci affected by barriers to gene flow having a reduced (or null) migration rate compared with the other category.
- **hetN:** a demographic model with heterogeneous effective population size across the genome. Heterogeneity was modelled in two possible ways: (i) the effective population size is beta distributed across the genome (Roux et al., 2016), (ii) two categories of loci have different effective sizes (Rougeux et al., 2017), with loci affected by background selection having a reduced effective size compared with the other category.

*Demographic parameters inferred for hetM models*. The studies we examined, and for which the estimates of the parameter values were reported, modelled heterogeneity in migration rates based on ‘two categories of loci’ that are interpreted as follows:

- ***m*:** migration rate among background loci averaged across directions of gene flow (i.e. from lineage 1 to 2 and from lineage 2 to 1).
- ***m***_**e**_: (reduced) effective migration rates among loci linked to gene flow barriers, averaged across directions.
- ***m***_**e**_ **/*m*** gives an estimate of the migration rate experienced by a fraction ***p*** of the genome relative to regions not associated with barriers to gene flow. Migration rate units do not matter as we analysed the ratio ***m***_**e**_ **/*m***.

***p***: proportion of the genome experiencing reduced effective migration rate.

The divergence process can be long and complex, with periods of geographical isolation alternating with phases of contact, making the traditional classification of speciation according to a simple geographical context insufficient (Abbott et al., 2013; Butlin et al., 2008). Instead, progress in the field would benefit from understanding the history of divergence and identifying periods of isolation and contact, the extent of gene flow (Sousa & Hey, 2013) and their impact on the evolution of reproductive isolation. In addition, because both drift and selection can drive divergence, understanding the neutral divergence history is not enough. The genic view of speciation (Wu, 2001) suggests that gene flow between diverging populations will be heterogeneous across the genome because the effects of barrier loci are only experienced strongly by closely linked loci until selection starts to overcome the impact of recombination (Barton, 1983; Barton & Bengtsson, 1986; Flaxman et al., 2014). Therefore, we need to understand how an initial set of genomically localized barrier loci expands into a genome‐wide barrier across the speciation continuum (Feder et al., 2012; Rafajlović et al., 2016; Stankowski & Ravinet, 2021; Wu, 2001).

These goals have become more accessible due to the development of multiple model‐based approaches using genomic data to infer demographic histories, accompanied by the increased availability of large population‐genomic datasets. Work using these approaches has confirmed that there are many examples of ‘speciation with gene flow’ (Pinho & Hey, 2010; Roux et al., 2016) in both the terrestrial and marine realms, spanning a range of taxonomic groups and environments (Faria et al., 2021; Potkamp & Fransen, 2019). By divergence/speciation with gene flow, we simply mean that there is at least one detectable phase of gene flow in the divergence process; gene flow does not have to be continuous throughout the whole process. It is not always clear whether divergence actually progressed during these phases of gene flow, but the crucial point is that gene flow did not completely erase divergence.

However, after more than 10 years of rapid progress in demographic modelling, there is not yet a synthesis of the available empirical studies to confront the ‘marine speciation paradox’ by assessing the geographical context in which speciation in the sea occurs. Therefore, it is a good time to take stock and ask: What have we learned about divergence and speciation in marine systems from these approaches? This is the aim of this review.

### 1.2 Demographic modelling

Sophisticated model‐based tools can infer the demographic history of divergence from genomic data, while simultaneously controlling for potentially confounding factors. The most widely‐used methods include Approximate Bayesian Computation (ABC; e.g. *DILS*: Fraïsse et al., 2021a), along with various tools that make inferences from the joint site‐frequency spectrum (e.g. *Fastsimcoal2*, Excoffier et al., 2021; δaδi, Gutenkunst et al., 2010; *Moments*, Jouganous et al., 2017). Although the details of these methods vary, they all involve comparing statistical summaries of empirical data with summaries of data produced under user‐defined demographic models. By modelling a range of different scenarios, it is possible to identify the one that fits the data best and obtain estimates for key biological parameters that describe the divergence process.

The models considered are usually variations of a simple two‐population model, where an ancestral population splits, giving rise to two descendant populations that diverge for *T* generations (see Becquet & Przeworski, 2007; Hey & Nielsen, 2004, 2007 for early development of these methods, Roux et al., 2016; Rougemont et al., 2017; Sousa & Hey, 2013 for more recent implementations and (Box 1) for an overview of methods discussed here). Common modifications to this base model include differences in the timing and symmetry of gene flow (often referred to as ‘migration rate’ and representing the movement of alleles) between the two populations, so that they resemble scenarios that are thought to be common routes to speciation (e.g. isolation‐with‐migration versus secondary contact (SC); Duvaux et al., 2011; Nadachowska‐Brzyska et al., 2013; Sousa & Hey, 2013). An important feature, under all scenarios, is the potential of these models to distinguish the effects of gene flow from the effects of lineage sorting. A major goal of demographic modelling is to distinguish between alternative classes of models, including those where gene flow occurred throughout the entire process (i.e. ‘isolation‐with‐migration’) and those where populations diverged during a period of allopatry before coming into contact again (i.e. ‘SC’). However, modelling also allows for more complex scenarios, e.g. including multiple phases of isolation and contact (i.e. ‘periodic connectivity’; Fraïsse et al., 2021b), along with other demographic changes (e.g. population size changes; Rougeux et al., 2017). Still, even the most complex models used are simplifications of reality, and a key limitation is that model fitting can only choose the best among the alternatives actually tested.

Genome scans, using differentiation statistics like *F*_ST_ (Wright, 1951), have found highly heterogeneous patterns of genetic differentiation along diverging genomes (e.g. Duranton et al., 2018; Nadeau et al., 2012; Stankowski et al., 2019; Tavares et al., 2018; Turner et al., 2005). These patterns are often assumed to reflect porous species boundaries: selection at barrier loci restricts gene flow locally in the genome (further promoting the build‐up of genetic differentiation at the barriers and close to them), while differentiation at ‘neutral’ loci is opposed by gene flow (Ravinet et al., 2017; Wolf & Ellegren, 2017). However, they can also reflect nonmutually exclusive processes other than the selection at (and linkage to) barrier loci, including background selection and sweeps at nonbarrier loci (Burri, 2017; Cruickshank & Hahn, 2014). This genomic heterogeneity is relevant for demographic modelling in two respects. First, because divergent and background selection can profoundly affect the genome, they can confound the demographic inference if not explicitly considered by the modelling approach. Second, if they are considered, demographic modelling can directly inform us about the effects of both types of selection on the genome. The distinction between these two functions is important: in speciation research, the aim is to control, as far as possible, the confounding effects of background selection in order to make inferences about the types of selection and barrier effects.

Critically, recent modelling approaches can include genomic heterogeneity in both the effective population size and in the rate of gene flow (Box 1). The former attempts to account for background selection (Charlesworth et al., 1993) and the latter to account for the effect of selection against migrants and hybrids (Barton & Bengtsson, 1986), including diverse mechanisms such as divergent selection or genetic incompatibilities, and prezygotic components of isolation, which have an effect similar to selection against heterozygotes (Barton & De Cara, 2009). These approaches are currently much simplified, for example, considering just two classes of loci, and so limited both in the control they provide for confounding effects and the insight they provide into selective processes. For gene flow, loci may be assumed to fall into different migration classes (Rougemont et al., 2017; Roux et al., 2016; Tine et al., 2014). In the early stages of speciation, these can be interpreted as: (i) those with a background migration rate (often interpreted as neutral regions and forming the majority of loci) and (ii) those with lower effective migration (interpreted as regions containing barrier loci and associated loci whose rate of gene exchange is reduced below the background rate). This is the interpretation we use here, but we note that the interpretation might change at later stages of speciation where most loci experience a strong barrier to gene flow, but some loci are still exchanged relatively freely between populations. Modelling gene flow heterogeneity can thus indicate the proportion of the genome affected by barriers to gene flow and the magnitude of the barrier effect.

Although demographic inference methods have now been applied to many individual case studies, syntheses of results from various species towards a more general understanding of the divergence and speciation process are needed. The potential of this comparative approach was highlighted in a study by Roux et al. (2016), who used ABC to fit a set of demographic models to 61 pairs of animal taxa with variable levels of genetic divergence. The most striking result was that variation in the probability of gene flow among the population pairs was most strongly correlated with the net genetic divergence (*D*_a_) between them. In Roux et al.’s study, marine taxa did not show a higher probability of ongoing gene flow when compared with terrestrial taxa, but the number of marine taxa included was too small to draw strong general conclusions about patterns of divergence in the sea.

In this review, we have compiled studies that have applied demographic inference methods to case studies of divergence in the marine environment, and we have synthesized their findings. This provides a broader survey for marine species than the study by Roux et al. (2016), and we also go further by comparing parameter estimates from the preferred models, particularly for heterogeneous gene flow. Which questions can we address with this synthesis of demographic modelling studies, focussing on speciation in the sea? We can quantitatively address the old question of how much evidence there is for divergence with gene flow (i.e. at least one phase of gene flow during the divergence process; Box 1), and whether this differs between phylogenetic groups or habitats. We can also ask how often divergence results from SC or strict isolation, indicating that geographical barriers do emerge in the sea, and how commonly do we find evidence for heterogeneous gene flow, i.e. for genomically localized barriers maintained in the face of gene flow. Finally, we can ask if the extent of genetic divergence between taxa relates to the probability of exchanging genes, and/or to the proportion of the genome experiencing reduced gene flow. We address these questions by synthesizing the results of published demographic analyses of marine speciation and highlighting how demographic modelling has changed and improved over time. We finish with a set of recommendations for reducing taxonomic and other biases and improving the reporting of results from demographic studies that will facilitate future meta‐analyses.

## 2 MATERIALS AND METHODS

To build our dataset, we examined all publications citing widely‐used methods for genome‐wide demographic inferences: Gutenkunst et al. (2010; δaδi), Jouganous et al. (2017; *Moments*), Excoffier and Foll (2011; *Fastsimcoal*) and Roux et al. (2016; ABC). In addition, a Google Scholar search was conducted (May 2021), using the following keywords: marine SNP ‘demographic model’. From that first list of papers, we only retained studies in marine systems providing demographic modelling of the divergence process, and where heterogeneity of gene flow across the genome was explicitly tested. We included pelagic, subtidal and intertidal benthic, anadromous, catadromous, salt‐marsh, lagoon and estuarine species. For each population/species pair, we recorded the results from the demographic inference (i.e. the method used, the preferred and second preferred model, the estimated parameters for the preferred model), differentiation metrics (*D*_a_: net divergence, Nei & Li, 1979; *D*_xy_: absolute divergence, Nei & Li, 1979; and *F*_ST_: relative divergence), taxonomic information and life‐history traits (Table S1). The preferred demographic model was decomposed into four categories: (i) the best demographic scenario (Box 1): SI, IM, AM, SC (which were tested in all studies) or PER (which was rarely tested), (ii) the presence/absence of heterogeneity in migration rates across the genome (hetM or not), (iii) or in effective population sizes across the genome (hetN or not) and (iv) the presence/absence of a temporal change in population size (in addition to the size change at the time of split). We contacted the authors to complete the dataset when metrics were not found in a paper.

Some methodological caveats need to be acknowledged and kept in mind when interpreting patterns. For example, we did not conduct a formal meta‐analysis, which would have required a reanalysis of the studies (since they used different demographic methods). We were also reluctant to apply statistical procedures to the final dataset. This is because many of the population pairs from the same study represent different pairwise combinations of the same set of taxa (e.g. with three taxa A, B, C, inferences were made for pairs A–B, A–C and B–C: Benestan et al., 2021; Cayuela et al., 2020), or, geographical replicates of the same species pair (e.g. with parallel evolution studies: Le Moan et al., 2016; Rougemont & Bernatchez, 2018; Stankowski et al., 2020), such that the results are nonindependent. In our analyses, we did not apply any phylogenetic or other corrections, meaning that the results may be impacted by the evolutionary relationships among the taxa and lack of independence among comparisons. In the following paragraphs, we discuss the relationships between inferred model parameters and estimates of genetic divergence and differentiation, mainly regarding what we might expect from biological predictions. However, it is important to acknowledge that some of these relationships may arise for technical rather than biological reasons. We discuss some possible technical artefacts below. Finally, the full dataset included missing values heterogeneously distributed across different variables; therefore, different data subsets were used to make the different graphs in our analyses. We limited ourselves to a narrative review for all these reasons, highlighting qualitative patterns.

## 3 RESULTS AND DISCUSSION

### 3.1 Demographic studies and the representation of biodiversity in the sea

**FIGURE 1:**
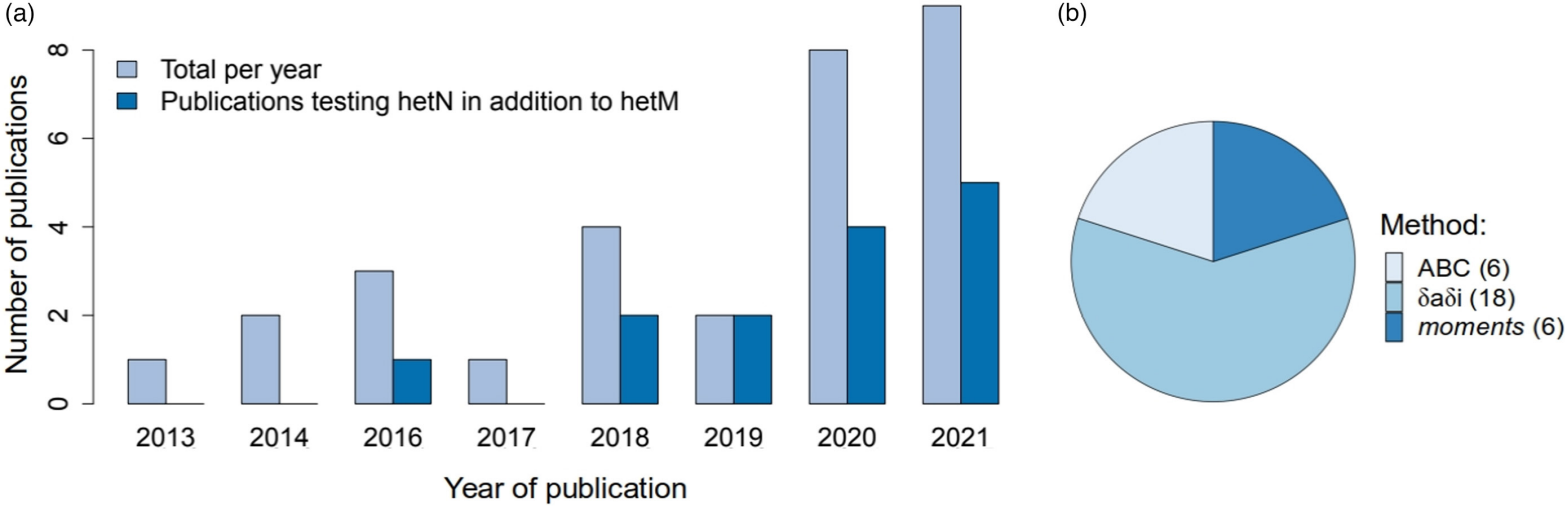
An overview of demographic modelling in the sea. (a) Increase in the number of publications (including hetM, and sometimes also hetN) in recent years (*n* = 30). (b) Distribution of the demographic methods used across publications (*n* = 30)

A total of 30 articles representing 116 pairs of population/species were retained in our final dataset. In some studies, multiple populations of the same taxon pairs were compared, so the 116 pairs of populations represent a total of 66 unique marine species. The number of studies modelling the divergence process and testing for hetM in marine organisms increased during the last 8 years, with most of the studies published in the past 2 years (Figure 1). Until 2018, hetN was rarely tested in the models and has been tested in a little more than half of the studies since then. δaδi was used in more than half of the studies and, together, the methods using a composite likelihood (CL) approach represented more than three‐quarters of the articles in our dataset. This may be because this approach is easier to implement, has effective online support and is less computationally intensive than ABC (Bourgeois & Warren, 2021). The outputs of the two main approaches (CL and ABC) differ in several ways, making a systematic comparison between the studies nontrivial or even impossible for certain aspects. For example, one of our goals was to check whether our dataset supports a relationship between variation in the probability of gene flow among the population pairs and the net genetic divergence (*D*_a_) similar to the one described by Roux et al. (2016). Unfortunately, this was not straightforward because most studies in our dataset used a CL approach and, therefore, did not provide posterior probabilities for contemporary gene flow. Moreover, hetN and hetM were modelled using two different categories of loci in studies using δaδi and *Moments*, whereas, in studies using an ABC approach, heterogeneity was modelled using a beta distribution for effective population sizes and migration rates.

Our data included species from nine phyla, with Chordata (represented mostly by species of bony fish) highly overrepresented. Chordata, Mollusca (mostly bivalves) and Cnidaria (mostly corals) together represented more than three‐quarters of the unique species in our dataset (Figure S1). One of the important aspects of studying speciation in the marine environment is the higher phylogenetic diversity compared with terrestrial environments, with some lineages only present in the sea (e.g. Echinodermata) and others that mostly contain marine species (e.g. Bryozoa, Cnidaria, Nemertinea, Platyhelminthes, Porifera, Haptophyta) (Grosberg et al., 2012; Guiry, 2012). This diversity provides unique study systems that might contribute to our general understanding of speciation. Among the seven lineages containing mostly marine species, four were represented in our dataset (Cnidaria, Echinodermata, Nemertinea and Haptophyta). However, we could not find any studies on macro‐algae or marine phanerogams, and our data only include two unicellular taxa (one species of Dinoflagellata and one Haptophyta) despite the ecological importance of the macro‐algae and phanerogams, and the abundance in the sea of the unicellular groups.

More than half of the 66 unique species were benthic (Figure S1). Coastal, pelagic and benthopelagic species were also well represented, whereas intertidal, catadromous and anadromous species were represented by eight taxa altogether. The biased habitat representation potentially deprives us of a general understanding of divergence and speciation since we might miss mechanisms specific to particular ecological contexts. For example, the intertidal habitat presents very strong environmental gradients that may underpin barriers, but this habitat is only represented by four taxa in our dataset.

This section shows that our dataset is biased in multiple ways. Consequently, a strict interpretation of the data allows no generalization to species or populations other than those included in the dataset.

### 3.2 Preferred demographic scenarios and the marine speciation paradox

**FIGURE 2:**
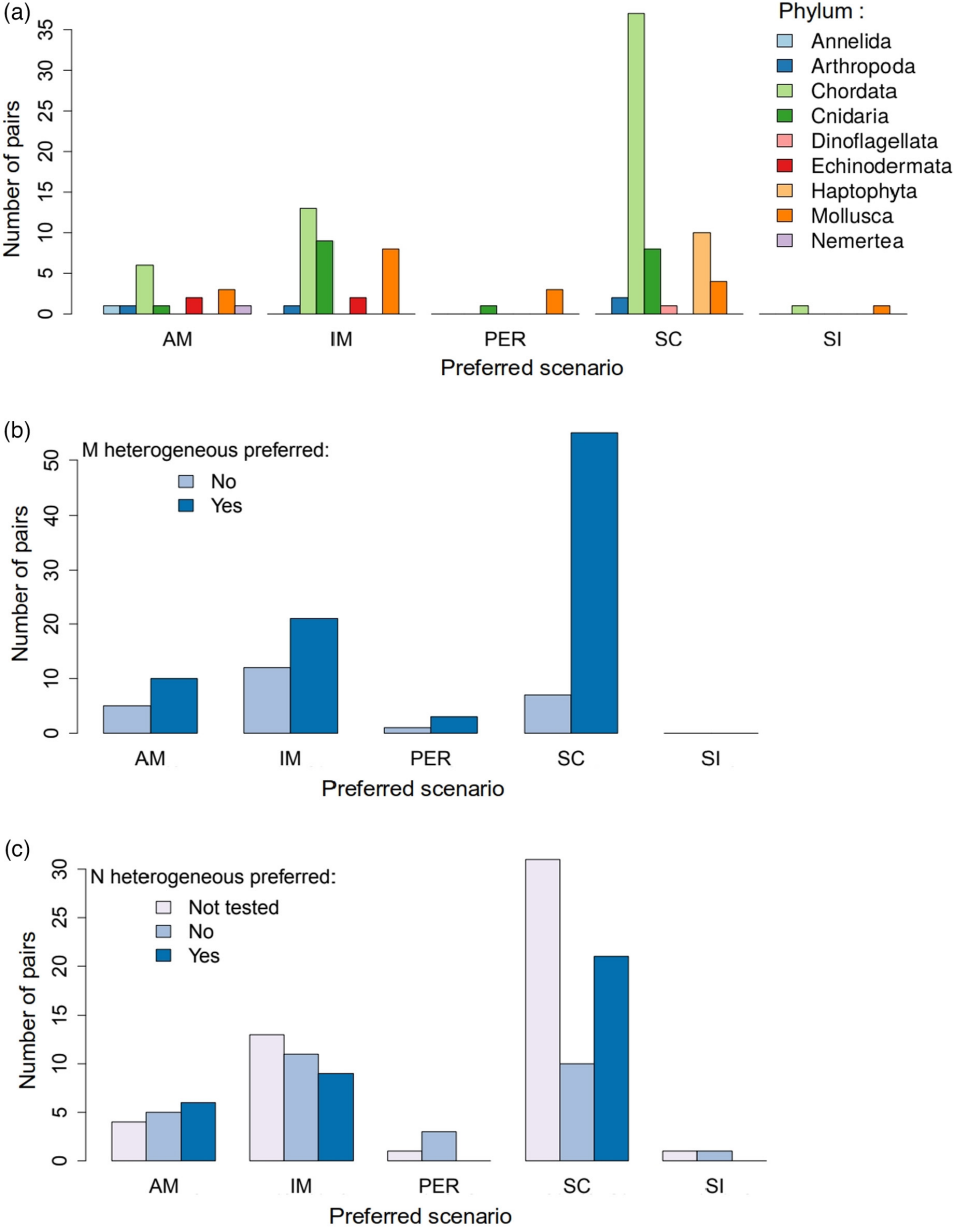
Preferred demographic scenarios in the sea. Distribution of the preferred demographic scenarios across all lineage pairs. Colours correspond to: (a) taxonomic phylum (*n* = 116), (b) hetM preference (*n* = 114), (c) hetN preference (*n* = 116)

Overall, in more than half (53%) of the 116 studied pairs, the SC demographic scenario received the strongest support. The isolation with migration (IM) and ancient migration (AM) scenarios was preferred in a substantial number of cases (28% IM and 13% AM), whereas the (periodic connectivity) PER and (strict isolation) SI scenarios were hardly ever the best supported (Figure 2).

The great majority of the preferred demographic scenarios involved periods without gene flow (all models except IM, i.e. 72% of 116 studies; Figure 2). This could be an artefact, perhaps due to the preferential fitting of more parameter‐rich models. However, if true, it clearly demonstrates that geographical barriers to gene exchange do exist in marine environments. Such barriers were inferred in all environments included in our dataset (except ‘marshes’ for which the single entry, for Atlantic silverside fish, was IM). Notably, among the species studied, those in benthic (69%) and coastal (54%) habitats were less likely to show evidence of geographical barriers than those in pelagic habitats (93%), where lower fragmentation might have been expected (although benthic and coastal species may have pelagic dispersal stages). Few currently allopatric species pairs (*n* = 9) are included in our dataset: these never have IM as the preferred model, but this makes only a small contribution to the overall pattern. The dispersal potential of study species might influence connectivity and so the preferred model. However, we do not have reliable estimates of dispersal for all cases. We do have data on adult body mass, which we expect to show a positive correlation with dispersal distance for organisms with active locomotion (Cloyed et al., 2021), despite known exceptions. We also have data on the size of the dispersal stage individuals (propagule size), where small size mostly represents small, feeding (planktotrophic) larvae with prolonged larval stages expected to disperse further. Small size is also likely to be correlated with a high propagule number, which might also result in greater connectivity. There was no apparent effect of adult body mass on the scenario preferred (Figure S2). For propagule size, the only clear pattern in our data was an excess of IM scenarios inferred for species with intermediate propagule size (Figure S2), but this was not robust to including heterogeneity in effective sizes (Figure S3, see below).

These observations suggest that marine divergence is not impeded by an open environment with few visible barriers, for two contrasting reasons: on the one hand, geographical barriers do exist and contribute to divergence, while, on the other hand, divergence can occur (or be maintained) despite phases of gene flow. Several examples of marine geographical barriers such as oceanographic currents (e.g. Rossi et al., 2020) or density differences between water masses (Hudson et al., 2020) are now well‐documented, and studies reporting divergence with gene flow are more and more common (e.g. Johannesson et al., 2020). Further interpretation of the numbers is probably unwise, given the unequal taxonomic coverage discussed above and the inevitable tendency to focus attention on taxa where there is some prior evidence for differentiation, particularly differentiation with ongoing gene flow. There may also be issues around the power to distinguish different scenarios: for example, the AM and SI scenarios may be challenging to separate, mainly if the period of ancient gene flow was short (e.g. Fraïsse et al., 2021a). Similarly, the SC model can be confounded with an IM model when the period of geographical isolation is short, or with an SI model at the other extreme when the contact is very recent. In cases of more complex demographic scenarios, for instance involving temporal variation of effective size across the history of the populations, a more parameter‐rich SC option might be preferred if it can account for features of the data that are unlikely under the simpler models. The effect of these unmodelled demographic events should not be ignored when studying population divergence, as they can lead to biases in model choice or parameter estimation (Momigliano et al., 2021).

Whole‐genome resequencing provides the most, and probably the least biased, information to compare demographic models. In our survey, WGS (22 cases) and RAD‐seq (57 cases) resulted in similar proportions of the main scenarios, suggesting no major issue with power in the reduced‐representation method (RAD‐seq) at this level. Cases that used RNA‐seq data (33 cases) were more likely to infer IM and, particularly, AM scenarios than SC. However, most of these results (*n* = 24) come from a single study (Roux et al., 2016), and most are based on the ABC approach (*n* = 27). Therefore, the reason for the discrepancy is unclear, but it indeed suggests caution and the need for simulation work to analyse biases associated with different data types and inferential approaches. Regarding the four remaining cases in our dataset, three were based on SNP chips and one on Sanger sequencing of target genes: all supported the SC scenario.

### 3.3 Genome‐wide heterogeneities during speciation

**FIGURE 3:**
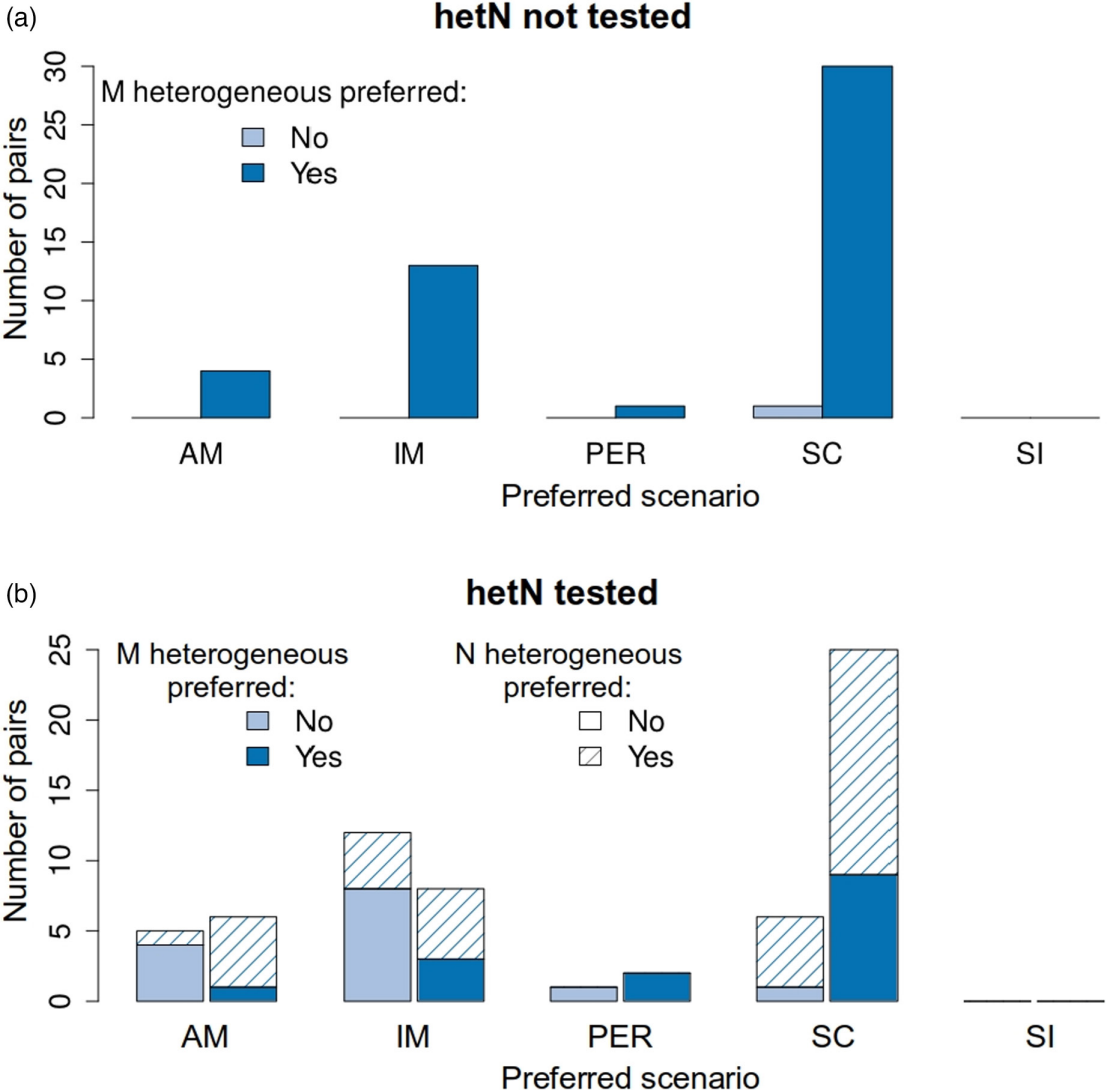
Preferred demographic models in the sea and genome‐wide heterogeneity in effective size. Distribution of the preferred demographic scenarios across all lineage pairs in studies where hetN was tested (*n* = 65, b) or not (*n* = 49, a)

A particular issue in the context of model complexity is the inclusion of heterogeneity in effective population sizes across the genome (hetN). Studies that test for hetN and include if it improves the model fit, have only recently become common (Figure 1). Therefore, a large fraction of the studied pairs in our dataset (43%, Figure 2c) were not tested for this type of heterogeneity. Failure to test for hetN might influence the preferred scenario: indeed, SC was more likely to be preferred (63% of 49 cases) when hetN was not tested than when it was (48% of 65 cases; Figure 3). This might be expected because SC can retrieve some of the patterns produced by changes in effective population size (Momigliano et al., 2021). In contrast, AM and IM were more likely to be preferred when hetN was tested (partly also reflecting a large number of cases from the study by Roux et al., 2016). These results agree with Roux et al. (2016), who showed that neglecting hetN tends to overestimate the prevalence of ongoing gene flow in the inferences (also discussed in Cruickshank & Hahn, 2014). Heterogeneity in effective size was detected in more than half of the cases where a test was made. This result could reflect variation in patterns of genome‐wide recombination and/or mutation rates across the studied taxa, but the failure to detect hetN may also reflect limited power in some studies. In studies where hetN was tested, the IM model was more commonly preferred for species in our survey with smaller propagule sizes (Figure S3), consistent with our prediction above, which was not met when tested across all studies. Future work should certainly include this test wherever possible. For the present, we decided to keep interpreting the results based on the whole dataset, highlighting cases where using only the studies in which hetN was tested gave different results.

Heterogeneous effective migration rates are expected during divergence, evolving from localized reductions around a few barrier loci towards strong, genome‐wide reduction in gene flow as speciation nears completion. We have only considered demographic analyses that tested for this heterogeneity (hetM). Allowing for hetM improved the fit of the preferred demographic scenario in 78% of the analyses (Figure 2b). However, if hetN is not also included in the model, this may increase the risk of inferring the presence of hetM where gene flow is actually homogeneous. Considering only the 65 cases where hetN was tested (Figure 3), the proportion of studies inferring hetM was reduced to 63%, suggesting that omission of hetN can, indeed, bias the results. One could argue that, like hetN, some degree of hetM is expected in all cases of divergence with gene flow: where it is not detected, this may again reflect a lack of power rather than an absence of heterogeneity. For example, when speciation is nearly complete, the genome‐wide barrier efficiently reduces gene flow across most of the genome, meaning that regions experiencing higher gene flow are rare and difficult to detect. Similarly, local adaptation of populations in similar environments will also generate heterogeneity in gene flow, especially where the number of locally‐adapted loci is small. However, it is unknown whether this sort of effect is detectable by demographic modelling. However, for hetM there may be exceptions where it is inferred but not related to genetic barriers: for example, homogeneous gene flow may be expected where the IM model is inferred for populations separated only by a geographical barrier, and this might also be true for SC after a short period of isolation.

Heterogeneous gene flow was more likely to be detected where SC was the preferred demographic scenario, whether hetN was tested (81%; Figure 3b) or not (97%; Figure 3a). Note that reduced‐representation methods (RAD‐seq or RNA‐seq) showed this effect (hetM in 39 of 42 cases where SC was inferred) more strongly than WGS (12 of 16 cases), arguing against a power issue. Technical artefacts are possible: for example, the SC model with hetM might be favoured if it explains patterns generated by demographic events that are not included among the scenarios fitted. If hetM is genuinely more prevalent under SC, it is not obvious why this should be the case. During a period of isolation, barriers are expected to accumulate randomly across the genome, without filtering according to effect sizes. This might result in initial heterogeneity that evolves towards a genome‐wide barrier to gene flow. However, following SC, gene flow might be expected to homogenize regions of the genome without strong barriers and produce an hetM genomic pattern. By contrast, under continuous gene flow (IM), divergence is only possible at loci under strong enough divergent selection to overcome gene flow, effectively filtering in favour of major‐effect barrier loci (Rafajlović et al., 2016; Yeaman & Whitlock, 2011). These loci are expected to have strong, but local barrier effects, generating greater heterogeneity of gene flow (although they could be missed by a sampling of loci, or not detected due to low statistical power). This suggests a pattern opposite to the one observed when hetN is tested (Figure 3b). Even so, the expectation of heterogeneity under the SC scenario would converge on the IM scenario with longer periods of contact (Ravinet et al., 2017): an excess of heterogeneity under SC is not expected in this argument. These arguments also suggest that heterogeneity should be least likely under the AM model: at the time when gene flow ceases, the situation is equivalent to IM, and subsequently, isolation should reduce any signal of heterogeneity. However, this is not the case; AM is slightly more likely to show hetM than IM (Figure 3). One potential hypothesis for this observation is that genetic incompatibilities are much more likely to arise in allopatry and so may contribute to greater heterogeneity of gene flow under the SC than the IM scenario. Significant incompatibilities can evolve on time scales of the order of 10^5^ generations (e.g. mice; Duvaux et al., 2011, grasshoppers; Shuker et al., 2005) that may be relevant to the cases analysed here. Thus, the frequent observation of hetM under the SC model may indicate a stronger role of incompatibilities than extrinsic barriers created by divergent adaptation in generating heterogeneity of gene flow.

The power to detect heterogeneous gene flow might vary with data types, inferential methods or divergence (see below). For example, Fraïsse et al. (2021a) showed that DILS could not detect barrier loci in the genomes of two populations that diverged very recently. Moreover, it is challenging to estimate separately the effects of heterogeneity in effective size (hetN) and gene flow (hetM) when both are actually present, as they can account for similar features of the data (e.g. they can both model genomic regions with reduced diversity resulting either from lower recombination rates or reduced gene flow). Further simulation work is needed to understand these methodological issues and clarify the biological expectations. We need to know the expected extent and distribution of genome‐wide variation in gene flow following various divergence scenarios to make meaningful tests of detection power.

### 3.4 The grey zone in the sea: proportion of barrier loci

When we compared divergence (*D*_a_ and *D*_xy_) with differentiation (*F*_ST_) between population pairs, we observed a stronger correlation between *F*_ST_ and *D*_a_ (Spearman correlation = 0.810) than between *F*_ST_ and *D*_xy_ (Spearman correlation = 0.463; Figure S4). This is expected since both *D*_a_ and *F*_ST_ are relative measures affected by levels of within‐population variation, contrary to *D*_xy_, which is an absolute measure of divergence (Cruickshank & Hahn, 2014). Because of the strong correlation between these different measures and since *F*_ST_ was more frequently estimated across studies, we focussed primarily on patterns in relation to *F*_ST_.

Although there was a slight tendency in our study to have some cases with lower *D*_a_, the range of differentiation (both *D*_a_ and *F*_ST_) represented in our results was similar to the one in Roux et al. (2016), suggesting that divergence/differentiation estimates are comparable (Figure S4). The different pairs covered a wide range of differentiation levels (*F*_ST_ from ~0 until almost 1), suggesting that the speciation continuum was fully represented, but more studies focussed on lower differentiation pairs. Interestingly, almost all pairs with very high differentiation (*F*_ST_ >0.5) corresponded to cases of divergence under the AM scenario, with two exceptions (one SC and one SI). In contrast, pairs diverging under the IM model had intermediate to low differentiation (0 < *F*_ST_ <0.5), whereas populations diverging under the SC model covered a wider part of the speciation continuum (0.025 < *F*_ST_ <0.75; Figure S4). It could be that the IM model is more likely to be fitted at low divergence for technical reasons. Nevertheless, the pattern suggests that, in a scenario with continuous gene flow (IM model), genome‐wide *F*_ST_ tends to be maintained at a relatively low level compared with other scenarios and that high differentiation is rarely achieved without an allopatric period. However, most models only provide information about the current or most recent period of gene flow, and some species currently isolated (AM and SI) might have initially diverged under an IM scenario. We would need estimates of the duration of divergence and possible earlier periods of gene flow to understand this better, but these were not consistently available. The wider range of *F*_ST_ values in the SC scenario is probably related to variation in the duration of the initial period of isolation and the time since contact, the proportion of the genome that is impermeable to gene flow due to the accumulation of reproductive barriers during that period, and/or the rate of migration upon SC.

**FIGURE 4:**
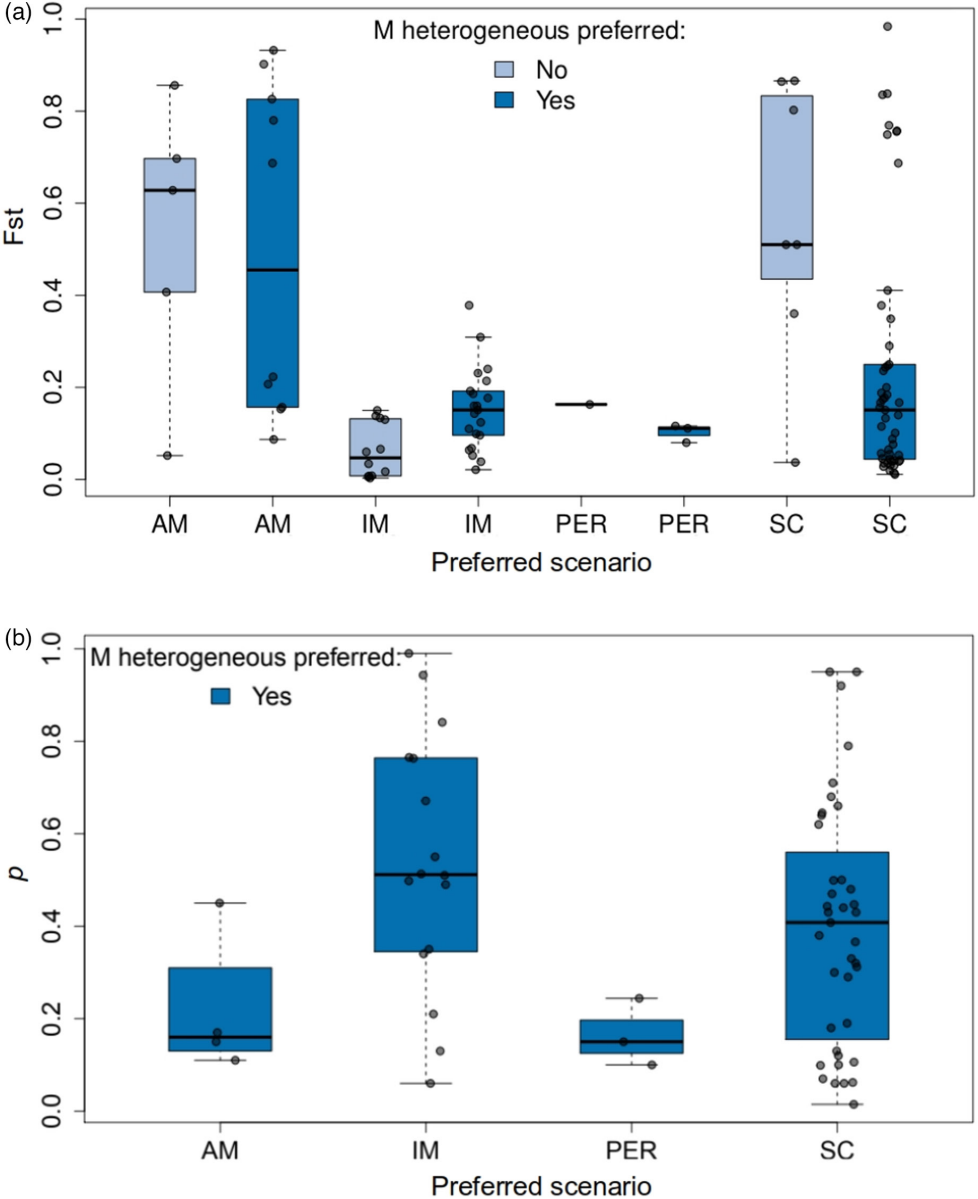
Distribution of (a) *F*_ST_ (*n* = 108) and (b) *p* (*n* = 62) across the preferred demographic models. For (b), only lineage pairs for which hetM was preferred are considered. *p* is the fraction of the genome with reduced gene flow

A wide distribution of *F*_ST_ values was observed in scenarios that included a period of isolation (SC and AM), with a tendency to be bimodal in studies where hetM was preferred (Figure 4a). The proportion of the genome experiencing reduced effective migration (*p*) under a hetM model was highly variable across studied pairs, encompassing the complete range from 0 to 1 (Figure 4b). Some pairs diverging according to the IM model showed low to moderate differentiation despite a very high proportion of the genome experiencing reduced gene flow, which might suggest widespread but weak reproductive barrier(s). Similar patterns were observed when using *D*_a_ or *D*_xy_ instead of *F*_ST_ (Figure S5).

**FIGURE 5:**
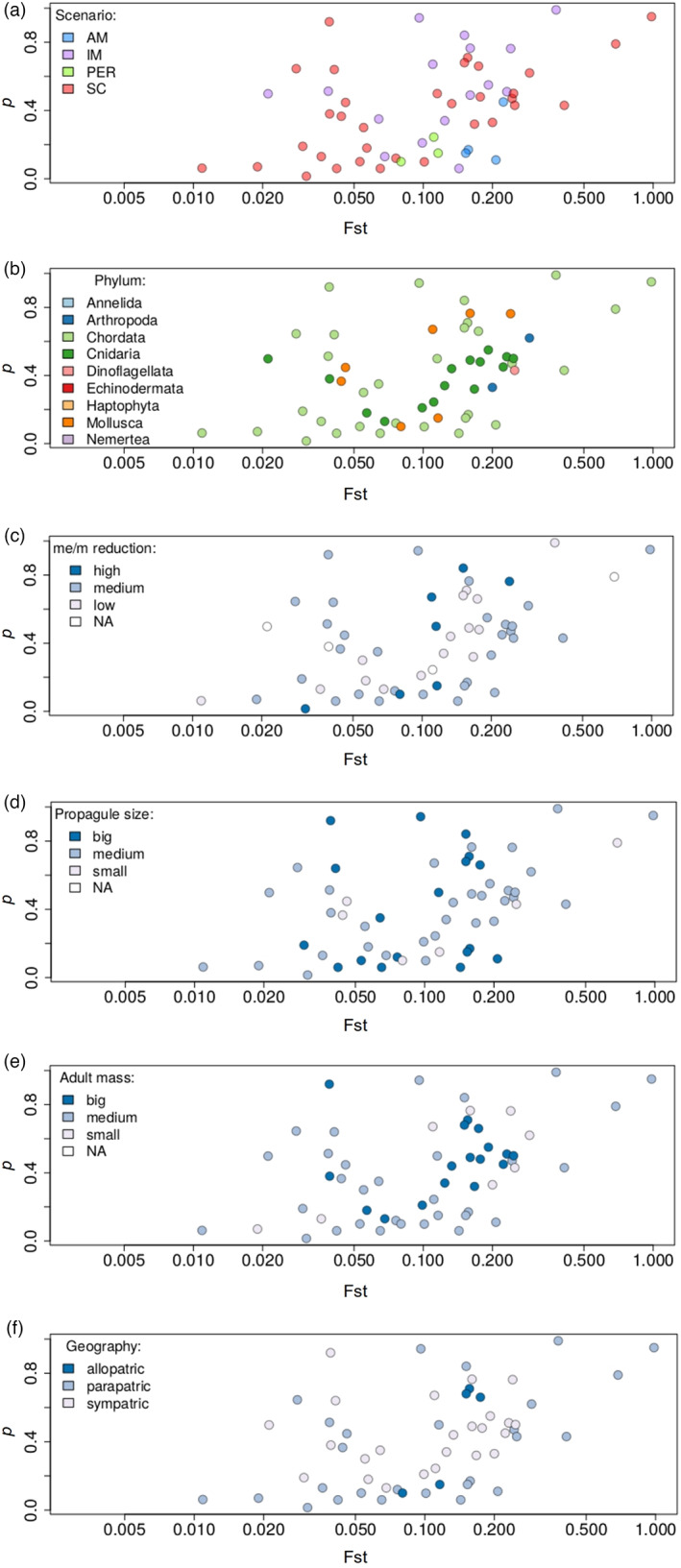
Correlation between *F*_ST_ and *p* (*n* = 56). *p* is the fraction of the genome with reduced gene flow. Colours correspond to: (a) preferred demographic scenarios, (b) taxonomic phylum, (c) categories of reduced gene flow based on quantiles of the ‘*m*_e_/*m*’ distribution, (d) categories of propagule sizes (i.e. the size of the dispersal stage individuals) based on the quantiles of the size distribution, (e) categories of adult mass based on the quantiles of the mass distribution and (f) geographical context of the populations studied. For the (c) panel: High reduction if the value is below the 25% quantile (0.021), low reduction if the value is above the 75% quantile (0.19) and medium otherwise. For (d) and (e) panels: Small if the value is below the 25% quantile (0.019 cm, 5.2 g), big if the value is above the 75% quantile (3.35 cm, 5 kg) and medium otherwise. In all panels, the *x* axis is in log scale

Focussing only on the population pairs where a model with hetM was preferred, we next examined the relationship between the maximum‐likelihood values of parameters that describe the migration rate (*m*, *m*_e_ and *p*; background migration rate, reduced migration rate in parts of the genome experiencing barriers and proportion of the genome influenced by barriers, respectively) and their corresponding estimates of *F*_ST_. If *F*_ST_ is at least partly indicative of the potential for gene exchange between populations, we might expect to see a positive relationship between the fraction of the genome experiencing reduced effective migration (*p*) and *F*_ST_. Figure 5 shows this expected positive relationship. However, it was quite weak (Spearman correlation = 0.416) and strongly influenced by the few points at the low and high ends of the *F*_ST_ distribution; the relationship was not so apparent for the range of *F*_ST_ values where most of the points were distributed (i.e. between 0.02 and 0.20). This suggests that *p* is a poor predictor of the level of genetic differentiation.

The weak relationship between *p* and *F*_ST_ may be due to the influence of other factors that can affect the level of genetic differentiation between populations or the accuracy of inferred parameters describing the pattern of heterogeneous migration. For instance, modelling the semi‐permeability to gene flow with only two categories of loci is perhaps not realistic and can inaccurately capture the fraction of the genome that resists gene flow. Contrary to this, there was no obvious effect on the other potential explanatory variables we examined (Figure 5), including the preferred demographic scenario (Figure 5a), taxonomic phylum (Figure 5b), propagule size (Figure 5d), adult mass (Figure 5e) and the geographical context (Figure 5f). Similarly, there was no obvious difference in the relationship between *p* and *F*_ST_ when considering only studies where hetN was also inferred (Figure S6, Spearman correlation between *p* and *F*_ST_ = 0.55). This suggests that the observed relationship is not strongly affected by the failure to include hetN as a model parameter, which might result in hetM being preferred to account for variation in the effective population size across the genome. Perhaps the most likely missing explanatory variable is the average genome‐wide level of gene flow, which we could not include because absolute estimates of *m*, *m*_e_ and *N* were unavailable from many studies (see Recommendations below).

We also examined the relationship between *m*_e_/*m*, and either *F*_ST_ or *p*. We focussed on these quantities because we might expect the magnitude of the reduction in gene flow to vary with the proportion of the genome experiencing that reduction. For example, when *p* is very high (i.e. when most of the genome is affected by barriers to gene flow), we might expect *m*_e_/*m* to be low, because the majority of the genome would be associated with barrier loci. Low *m*_e_/*m* ratios might not be easily captured when most of the genome is affected by barriers to gene flow because, in this case, hybridization will have very little effect even on neutral regions (i.e. most introgressed ancestry will be removed by selection). A measure of the frequency of interspecific crosses would be needed to estimate the expected value of *m* in the absence of barriers. In contrast, there are no explicit predictions when *p* is low, because the few barrier loci might exert strong or weak effects resulting in wide variation in *m*_e_/*m*. Also, note that the migration rate expected for barrier and nonbarrier loci is equal with *p* = 0 and *p* = 1, but in these cases, *m*_e_ is not estimated because models without hetM would be preferred. Overall, we observed no clear association of *m*_e_/*m* with *p* or with *F*_ST_ (Figure S10).

### 3.5 The grey zone in the sea: position on the divergence continuum

**FIGURE 6:**
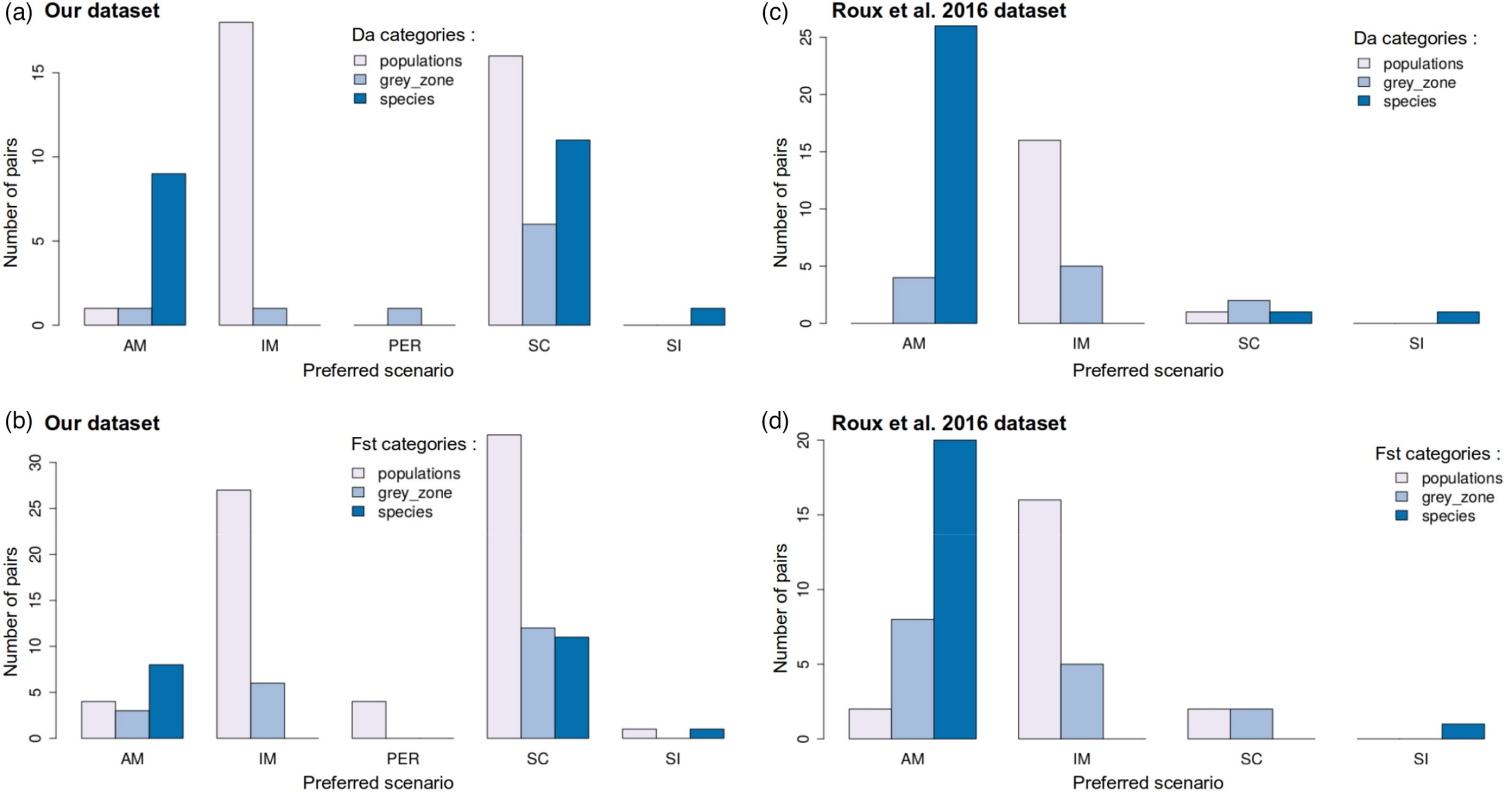
Preferred demographic models along the speciation continuum in the sea. Distribution of the preferred demographic scenarios across all lineage pairs. Colours correspond to three categories of divergence, defined in Roux et al. (2016), either from *Da* values (a: *N* = 65, c: *N* = 56) or *F*_ST_ values (b: *N* = 110, d: *N* = 56): (i) populations: *Da* below 0.5% or *F*_ST_ below 0.19; (ii) species: *Da* above 2% or *F*_ST_ above 0.56; (iii) grey zone: Values in‐between. Panels (a) and (b) refer to the dataset we analysed (Table S1), while panels (c) and (d) refer to Roux et al. (2016)

We next considered our results in the context of the study by Roux et al. (2016), using their thresholds to categorize the studied pairs along the speciation continuum. Specifically, we defined the taxa in each studied pair, with either *D*_a_ (Figure 6a, based on the threshold defined in Roux et al. (2016) in Figure 1) or *F*_ST_ (Figure 6b, based on the threshold defined in Roux et al. (2016) in Figure S6A), as being (i) populations from the same species when *D*_a_ <0.5% or *F*_ST_ <0.19, (ii) two species when *D*_a_ >2% or *F*_ST_ >0.56 or (iii) within the ‘grey zone of speciation’ for divergence values in‐between. By doing this, we were able to evaluate the contribution to the speciation grey zone of each inferred demographic scenario in our dataset. As expected, we found that the AM scenario was inferred more often in pairs with *D*_a_ expected from fully isolated species, while IM was generally found between taxa whose *D*_a_ was expected from populations of the same species. In contrast, SC was inferred in a range of taxa spread over the entire continuum (Figure S6A). Comparable patterns were found when using *F*_ST_ to categorize the studied pairs (Figure S6B) and were already described above (Figure S4). A similar contribution of the different scenarios was found in Roux et al. (2016), although many fewer SC scenarios were inferred in their study (Figure 6cv‐sf). Therefore, scenarios with expected current gene flow (SC and IM) were more commonly inferred between pairs with a *D*_a_ expected from population differentiation or between taxa falling in the speciation grey zone than between pairs with a *D*_a_ in the range expected from two species (Figure S7).

HetM was more likely to be inferred between pairs from the speciation grey zone, which is expected as it is the critical area where speciation is in progress, and so, the barrier to gene flow is potentially semi‐permeable (Figure S8). In contrast, hetN was frequently inferred along the whole speciation continuum, although it was less likely in the most divergent pairs, with divergence values at the level expected from fully isolated species (Figure S9). There is no obvious reason to expect these differences among divergence categories as hetN should capture the effect of background selection at linked sites, which is expected during the whole divergence history. Still, this pattern was consistently found here and in Roux et al. (2016). One possible explanation could be that the extent of differentiation is more homogeneous in the most divergent pairs (Figure S8), therefore it may be easier to reproduce the data with a simple model without any genomic heterogeneity included (Figure S9).

Surprisingly, evidence of gene flow was found in some studied pairs that are likely to be fully isolated (Figure S7) based on the 2% *D*_a_ threshold defined in Roux et al. (2016). While these contrasting results should be discussed cautiously, one possibility to explain this mismatch is that the speciation grey zone in the sea spans over a larger range of *D*_a_ than earlier suggested (Roux et al., 2016). However, most of the studies reviewed here were based on RAD‐seq or WGS, while the speciation grey zone in Roux et al. (2016) was defined from coding data obtained from transcriptome sequencing. Overall, this inconsistency calls for more fine‐tuning of the speciation grey zone (Galtier, 2019), either by extending the number of pairs to refine its delimitation or by evaluating the robustness of the *D*_a_ threshold inferred from the noncoding part of the genome.

## 4 CONCLUSIONS AND RECOMMENDATIONS

We have reviewed 10 years of demographic modelling in the sea to improve our understanding of the divergence and speciation processes. Our study revealed interesting patterns but also encountered roadblocks that limit its outcomes. Here, we summarize our key findings and make recommendations for future studies.

When confronting the marine speciation paradox, most of the demographic scenarios best describing the divergence process included a period of isolation, suggesting that geographical or physical barriers to gene flow are indeed present in the sea. On the other hand, divergence processes due to strict isolation were scarce, suggesting that divergence mostly occurs with gene flow for at least part of the process.

Heterogeneity of gene flow (hetM) along the genome was detected in the majority of pairs, but this proportion was affected by whether heterogeneity of effective population size (hetN) was tested or not. When considering only pairs where both hetN and hetM were tested, hetM was still detected in most cases but in a smaller proportion. Heterogeneous gene flow was more likely to be detected where SC was the preferred demographic scenario, but there are multiple possible interpretations for this pattern, including biological and technical effects. Some possible biological reasons were discussed by Duranton et al. (2018).

We found a weak correlation between the proportion of the genome experiencing reduced gene flow (*p*) and *F*_ST_. The weakness of this relationship might be because *F*_ST_ is affected by other factors such as phylogenetic position or geographical context of the studied pairs, but there was no clear effect of those on *F*_ST_. Similarly, we did not see any evidence for a link between the reduction in gene flow at barrier loci (*m*_e_/*m*) and *F*_ST_. In order to understand these relationships more fully, it would help if studies reported *m*, *m*_e_ and *N* in consistent ways, preferably on untransformed scales, to allow for estimating the effect of average gene flow. However, it should also be noted that estimating *m* in demographic models is problematic because it will always include the genome‐wide reduction in gene flow due to barriers and therefore reflect a background *m*_e_ rather than the actual migration rate. Absolute estimates of divergence times (in generations, estimated separately from *N*_e_) would also be valuable, but many studies only report scaled values.

Generally speaking, the position of the grey zone of speciation on the divergence continuum was similar in our dataset to the values in Roux et al. (2016). We found that pairs with a preferred demographic scenario implying current gene flow (SC, IM) were found in higher proportions in the ‘populations’ part of the divergence continuum and in the grey zone. In contrast, pairs with a preferred demographic scenario implying no current gene flow (SI and AM) were found in higher proportions in the ‘isolated species’ part of the divergence continuum. We also found pairs for which gene flow was still present despite having a differentiation level higher than the upper limit of the grey zone defined in Roux et al. (2016), suggesting that the grey zone of speciation is actually wider than previously thought.

Below, we make recommendations to improve our general understanding of marine speciation (but also applying more generally to demographic modelling) and to support future possibilities for new syntheses and more detailed meta‐analyses.

1. Aim for a balanced representation of marine taxa, environments and the divergence continuum.

Our study showed that both phylogenetic and habitat representations are highly biased. In particular, major groups such as Echinodermata, Bryozoan, Cnidaria, Nemertinea, Platyhelminthes, Porifera, Haptophyta, macro‐algae, marine phanerogams and unicellular taxa were very few or absent in our dataset. Similarly, some habitats were highly under‐represented (e.g. the intertidal zone, although this is, however, rather species‐poor). We argue that correcting this bias would benefit our general understanding of the divergence process in the sea (and in general across environments). Moreover, studies on late stages of speciation were less represented, and this is probably because studies on speciation tend to focus on semi‐isolated species, therefore putting a lot of emphasis on the grey zone (Kulmuni et al., 2020). We argue that to better understand the process of divergence and speciation, we should cover the entire continuum in as unbiased a manner as possible. Therefore, we recommend that future studies cover a wider range of divergence levels and include lineages that are believed to be more strongly isolated. This will facilitate a better understanding of the whole speciation process. For example, (i) does the grey zone of Roux et al. (2016) differ among taxa and environments, (ii) is the grey zone wider if defined by the rate of gene flow or the proportion of the genome experiencing a barrier rather than the probability of ongoing gene exchange and (iii) what demographic histories have been experienced by lineages that now show very strong or complete reproductive isolation?

2 Standardize sampling designs, where possible.

Here, an additional layer of complexity was the lack of standardization of sampling design across the different studies. In some cases, demographic inferences were performed between populations directly in contact, while in other studies, populations were only connected indirectly via other structured populations. In the latter situation, it is difficult to see how contemporary patterns of connectivity in a metapopulation might influence the inferences of historical gene flow in a given pairwise comparison between distant populations. Similar problems applied to the comparative work of Roux et al. (2016), where the studied pairs encompass various geographical situations. Ideally, future work should consider standardizing the sampling strategy across the studied taxa, as Gagnaire (2020) suggested. For this purpose, multi‐species contact zones (‘suture zones’) represent an ideal framework to further characterize the role of the divergence history in speciation among many pairs of populations sampled over a shared spatial and environmental scale (Johannesson et al., 2020). Where standardization is not possible, clear reporting of the geographical context (distance, intervening populations, physical barriers, etc.) is important.

3 More consistent application of demographic modelling methods.

A significant challenge in our dataset was the heterogeneity in the inferential methods used for demographic modelling (ABC, δaδi, *Moments*). The different methods produce outputs that are not directly comparable (likelihood vs Bayesian‐based) and model key processes differently (e.g. hetM was modelled with a distribution of migration rates versus using only two classes of rates), making formal comparison difficult or impossible. Another difficulty reflected in this study is that, while Roux et al. (2016) used synonymous divergence based on transcriptome sequencing data, most studies estimated divergence based on RAD‐Seq or WGS, making the comparison difficult. In the absence of a gold‐standard method, applying multiple methods (or at least one likelihood and one Bayesian‐based) on the same dataset and the same set of models would be valuable. This would ensure that studies were more comparable and would also help confirm the choice of the best model in a particular study using goodness‐of‐fit tests.

4 Toward more complex demographic models.

Regardless of the method chosen to conduct the demographic analysis, we strongly recommend modelling both the heterogeneity of population size and the heterogeneity of gene flow along the genome, as ignoring either can impact the preferred demographic scenario obtained. We further encourage the community to improve the modelling of these processes beyond two‐class models (for example, using continuous distributions of Ne and Me as in Roux et al., 2016) to shed light on the underlying selective processes. The field is progressing toward these more realistic models (Johri et al., 2022), especially to infer jointly the demography and the effect of background selection in one‐population scenarios (Johri et al., 2020).

Similar biases in model choices were reported recently by Momigliano et al. (2021) when not accounting for variation in population size prior to the population split or in the daughter populations. Allowance for such population size changes was only included in two of the studies reported here, so its consequences could not be evaluated. We recommend testing one‐population scenarios to help identify specific demographic events and then modelling more realistic two‐population divergence scenarios.

As computing performance improves, solutions to more complex models can be reached in acceptable times, pushing for more realistic models to be implemented. For example, a GPU version of δaδi was recently published (Gutenkunst et al., 2021), allowing the exploration of models with up to five populations. These multi‐population models will likely be relevant to study the demographic history when multiple semi‐isolated taxa interact in nature, such as in cases of parallel evolution (e.g. Butlin et al., 2014; Le Moan et al., 2016; Stankowski et al., 2019) or species complexes (e.g. Benestan et al., 2021; Cayuela et al., 2020; Fraïsse et al., 2021b). This added realism has benefits, but it may also make comparisons among studies more difficult. Moreover, with more complex models, there is a risk of overfitting aspects of the data that the demographic model still could not capture. To minimize such risk, we recommend comparing nested models of increasing complexity to identify the most critical evolutionary components of divergence (see Rougeux et al., 2017 and Fraïsse et al., 2021a for examples of such procedures).

With whole‐genome sequencing data, the consideration of genomic variation in recombination rate is crucial for inferring the demography and the selective processes involved during divergence. But current methods assume independence between SNPs (e.g. δaδi or *Moments*) or between genomic blocks (e.g. DILS or gIMble [https://github.com/DRL/gIMble]) to compute likelihoods or posterior probabilities accurately. Therefore, data pruning based on linkage disequilibrium has become good practice for demographic inferences, despite the loss of information. ABC‐based methods like DILS can explicitly consider intra‐locus recombination in the model; however, due to excessive computing time, this method cannot be applied to whole chromosomes. Unlike classical ABC approaches, Convolutional Neural Networks (CNN) do not require summary statistics and require fewer simulations for model comparison and parameter estimation (Flagel et al., 2019). Thus, it should soon be possible to treat a chromosome as an image where selective and/or demographic events have left footprints that the CNN will learn to recognize whilst considering the recombination rate along the chromosome.

5 Clearer reporting of results.

It was sometimes difficult to extract the parameter values estimated for the best model from the published results. For example, occasionally, there were conflicts in how *m* and *m*_e_ were reported between the two directions of gene flow. Avoiding confusion on this particular issue may involve replacing *m*_e_ with a parameter that directly quantifies the extent to which *m* is reduced in barrier regions (e.g. Momigliano et al., 2021). More generally, we encourage authors to take particular care in reporting the units for the parameter values estimated, and to be precise about whether (and how) the values have been transformed into meaningful biological units. It would greatly aid comparative work to have estimates of divergence times, *N* and *m* (and *m*_e_), rather than scaled values. With WGS data accumulating, it should become possible to estimate mutation rates (and their variability) for more systems, making these unscaled values more accessible. A public database, with a consistent reporting format, would be ideal.

The net synonymous divergence, *D*_a_, appears to be the best metric to define the grey zone of speciation (Roux et al., 2016). However, *D*_a_ and *D*_xy_ were not reported for most of the studies we gathered (although we could obtain more of these metrics by contacting the authors). In the end, *F*_ST_ was the differentiation metric obtained for most studies, and that is why we ended up using it to scale the differentiation continuum in our study. *D*_a_ and *D*_xy_ are more difficult to estimate than *F*_ST_ with RAD‐seq data or other reduced‐representation approaches, but this will change with the increased use of WGS. We argue that all three divergence/differentiation metrics should be systematically reported in studies modelling the divergence process. Furthermore, an annotated genome can be partitioned into different functional categories (e.g. coding vs noncoding regions and synonymous vs nonsynonymous sites) to estimate divergence under different selective regimes (e.g. *dN* versus *dS*) based on larger genomic regions than RAD‐seq data.

6 Conduct simulation studies to rule out technical explanations for general patterns.

Empirical studies should systematically conduct simulation work to assess the conditions in which the inferential method(s) used can correctly distinguish between the models, and reliably estimate parameter values given the data. For example, it will be crucial to simulate divergent selection and background selection (with varying distributions of selection coefficients) under various demographic scenarios and then test whether these patterns are appropriately captured by hetM and hetN such that the two selective processes can be distinguished. This will also help identify under which (species‐specific) conditions a set of demographic models tested can potentially be reduced to less complex models (e.g. accounting for only hetN instead of both hetN and hetM) in order to, for example, reduce power issues due to parameter‐rich models (see recommendation 4) or to reduce the computational costs. However, as such knowledge is currently lacking, we encourage researchers, whenever possible, to include both hetN and hetM in demographic modelling as this will help us understand how the relative importance of the different processes contributing to the heterogeneity of gene flow in the genome may change along the speciation continuum (see also recommendation 4).

Moreover, the interpretation was not trivial for some of the results obtained here, because we lacked clear baseline expectations. In some cases, we used qualitative expectations, but in others, we were guided by speculations. For example, hetN was not systematically detected in our dataset, and we argued that this might reflect a power limitation. However, this remains to be tested using simulations. In general, we argue that an in‐depth interpretation of the results and patterns observed requires rich quantitative expectations (e.g. correlations between demographic parameters and biological metrics such as divergence estimates).

7 Move toward more integrative studies.

Moreover, future studies should aim to connect demographic model estimates with biological features of the organisms studied, such as dispersal capacities and reproduction modes, and to estimates of reproductive isolation and the relative contribution of different reproductive barriers across the speciation continuum. For taxa that form hybrid zones in nature, genomic variation in introgression in the zone can be used as an alternative way to determine the proportion of the genome under selection and to identify specific barrier loci. Combining these different data types will be essential for understanding the drivers of speciation rather than simply documenting patterns of divergence. Similarly, it will be important to combine demographic modelling with information about the oceanographic history, e.g. to associate phases of allopatry with historical dispersal barriers. In some cases, niche modelling approaches or the fossil record may inform about historical distribution ranges.

We believe that demographic modelling, particularly based on WGS data (Smith & Flaxman, 2020), has a great potential to provide insight into divergence and speciation. This potential is already being realized for individual case studies, but the field of speciation research also needs to seek generalizations. Making the most of demographic modelling in this integrative context requires comparative analysis. Our study adds to the small number of existing attempts in this direction and demonstrates the real promise of the approach. However, it also shows the difficulties to be overcome. We hope that these recommendations will lead to much more powerful and insightful analyses in the future.

## CONFLICT OF INTEREST

We have no conflict of interest to declare.

## Supporting information


Figures S1‐S10

Table S1

## Data Availability

Data sharing not applicable to this article as no datasets were generated or analysed during the current study.
