# Supplementary material for: Ten years of demographic modelling of divergence and speciation in the sea
Source: Evol Appl. 2022 Jun 26;16(2):542–59. doi: 10.1111/eva.13428 (PMC9923478; doi:10.1111/eva.13428)

**Supplementary figure 1. Features of the dataset.** A) Representation of the taxonomic phylum in the sea (data from Grosberg et al., 2012 and Guiry, 2012). B) Distribution of the taxonomic phylum across the taxa studied (n=66). C) Distribution of the taxonomic classes across the taxa studied (n=66). D) Distribution of the adult habitat across the taxa studied (n=66). Brown, red and green algae were grouped into a single clade; algae include freshwater and terrestrial species, but most are marine. Plants, which include about 100 marine species, are not represented. Phyla with 90% or more marine species are: Bryozoa, Cnidaria, Echinodermata, Haptophyta, Nemertea, Platyhelminthes, Porifera.


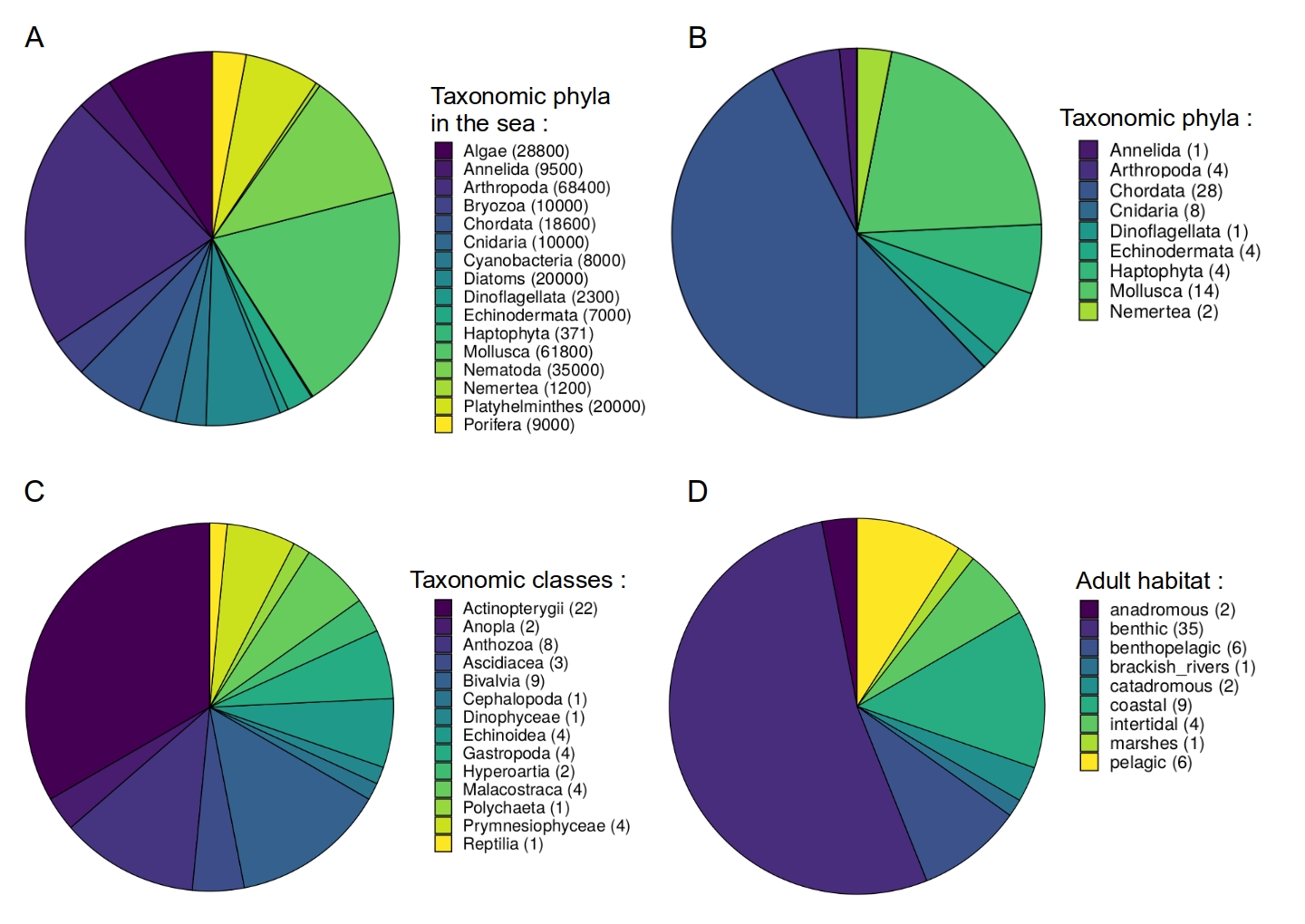


**Supplementary figure 2. Preferred demographic scenarios in the sea.** A) Categories of propagule sizes (i.e., the size of the dispersal stage individuals) based on the quantiles of the size distribution (n=115). B) Categories of adult mass based on the quantiles of the mass distribution (n=114). C) Geographic context of the populations studied (classification in the sympatric vs parapatric category was sometimes tricky, n=116). For A) and B) panels: small if the value is below the 25% quantile (0.019 cm, 5.2 g); big if the value is above the 75% quantile (3.35 cm, 5 Kg); and medium otherwise.


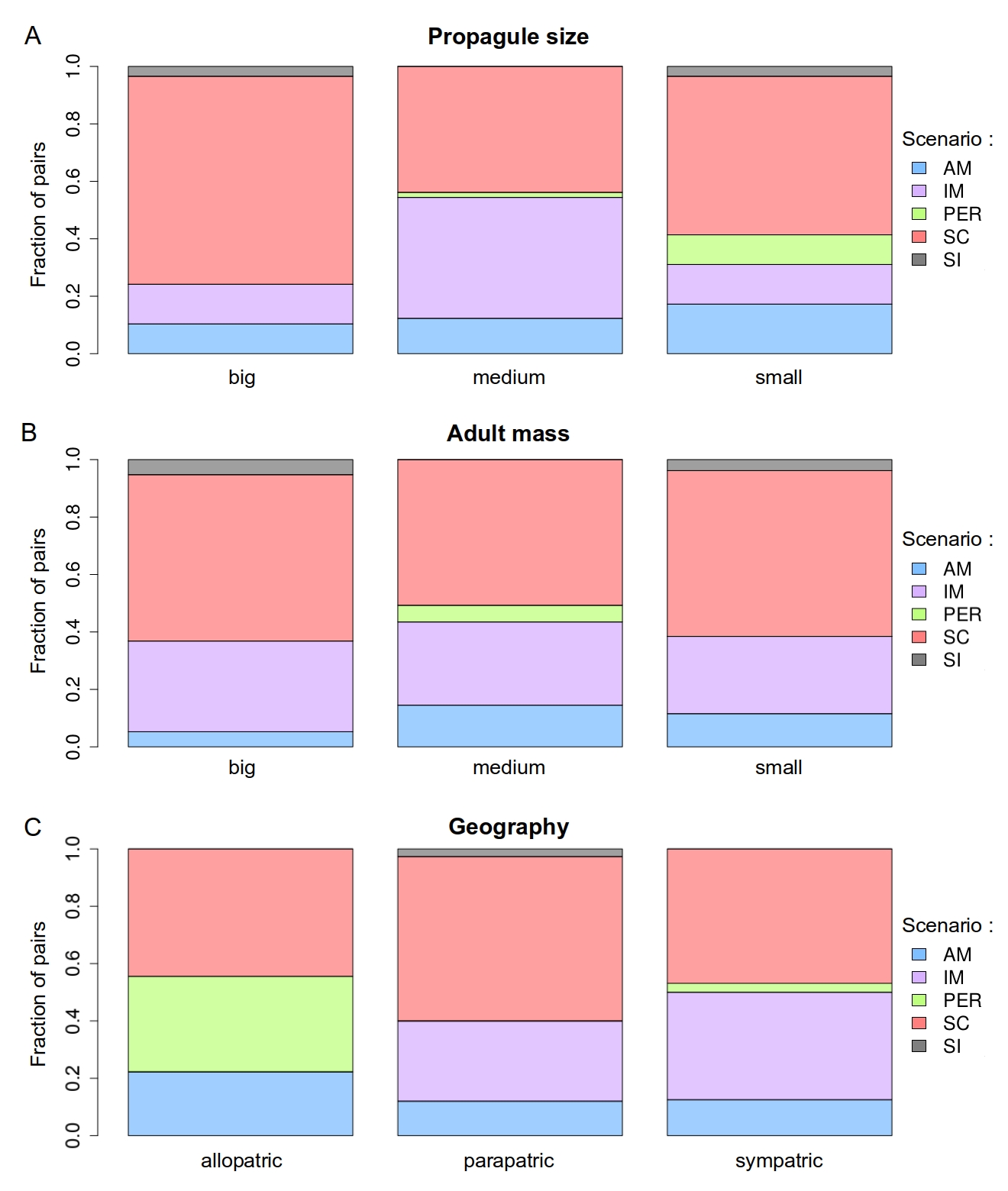


**Supplementary figure 3. Preferred demographic scenarios in the sea.** Studies where hetN was not tested were excluded. Other details match Supplementary figure 2, except sample sizes: A) n=65, B) n=64, C) n=66.


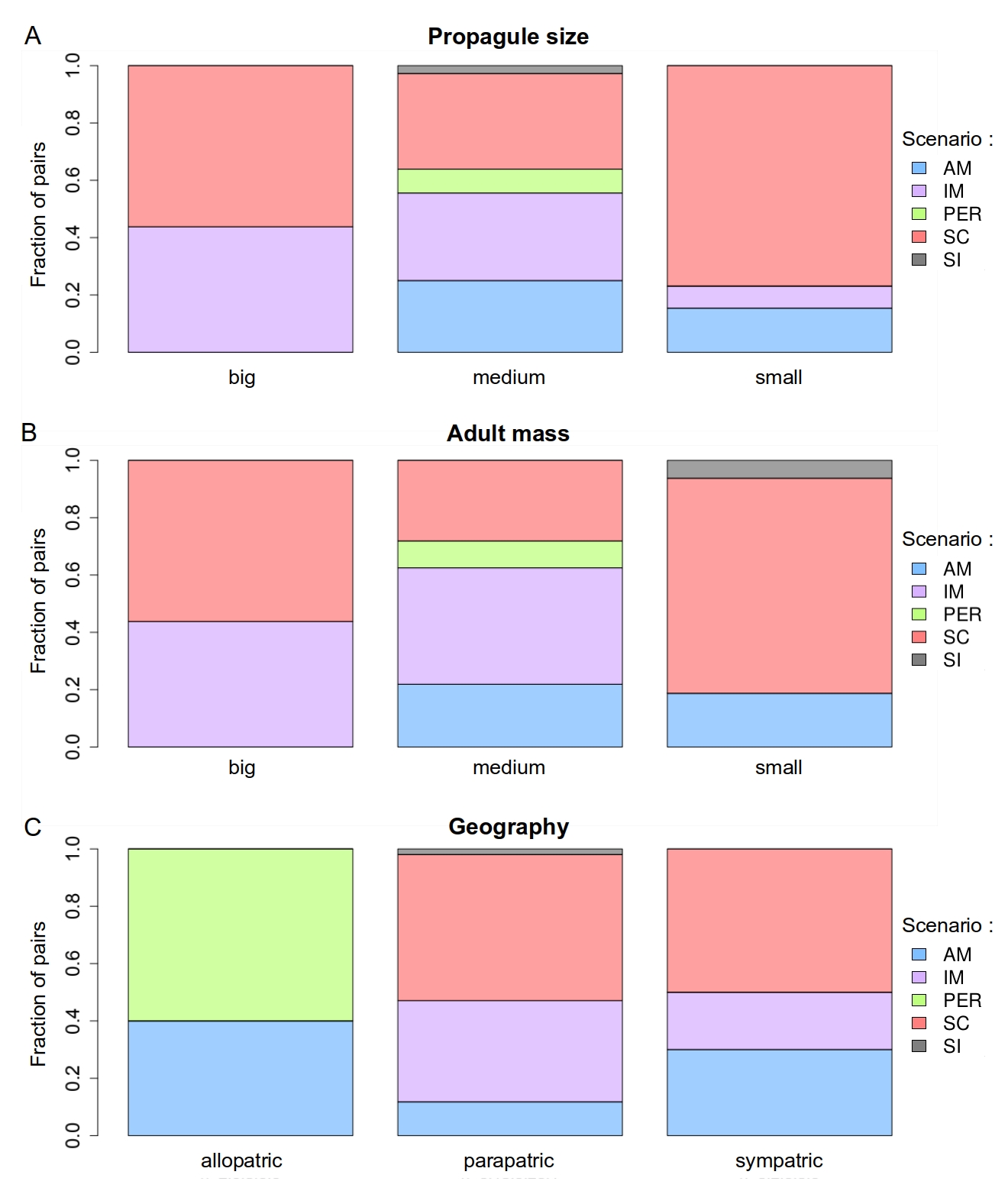


**Supplementary figure 4. Correlation between divergence/differentiation metrics.** Correlations between *F_ST_* and *Da* (A, n=61) or *Dxy* (B, n=61) were plotted across all lineage pairs. Studies in which metrics were deduced by the formula *F_ST_* =*Da*/*Dxy* were excluded. Small grey dots correspond to the animal pairs in Roux et al., 2016 (excluding the panmictic pairs). Colours correspond to the preferred demographic scenarios. In all panels, the x and y axes are in log scale.


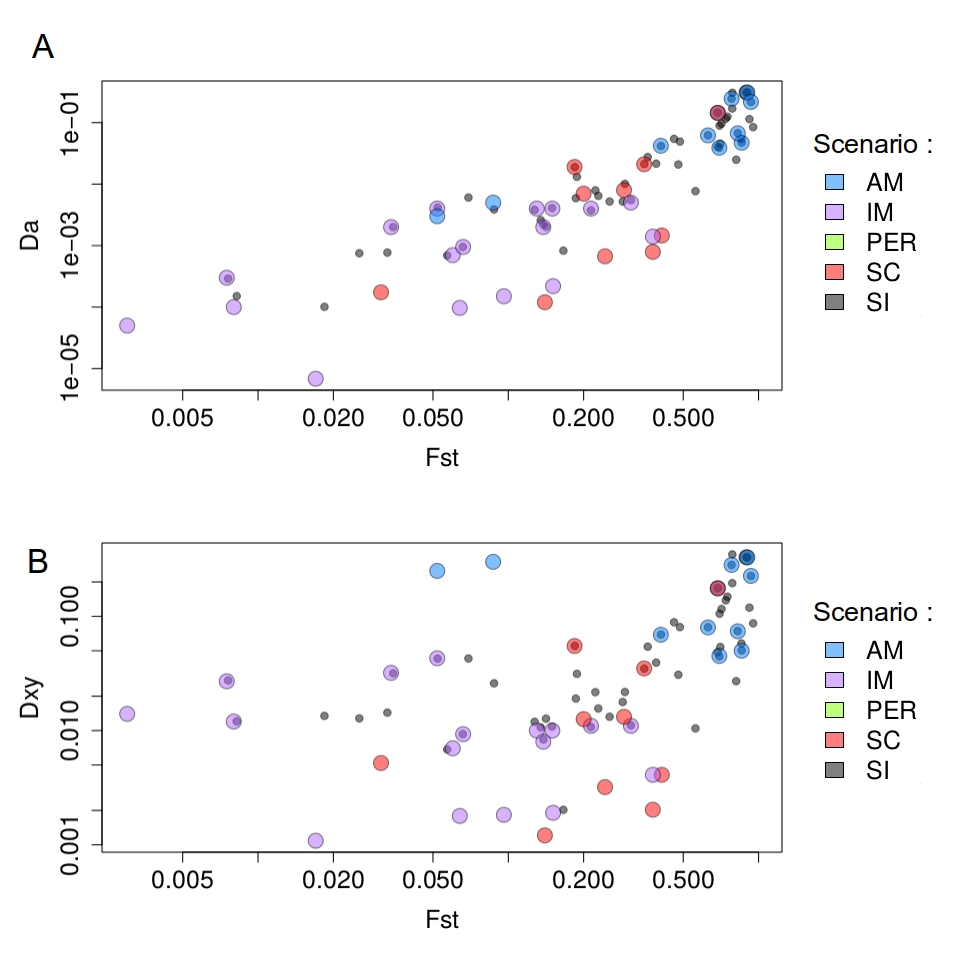


**Supplementary figure 5. Distribution of (A) *Da*** (n=64)**, (B) *Dxy*** (n=64) **and (C) *m_e_/m*** (n=62)**.** For (C), only lineage pairs for which hetM was preferred were considered. The y axis in (C) represents the ratio between the effective migration rate at loci linked to gene-flow barriers (*m_e_*) and the background migration rate (*m*).


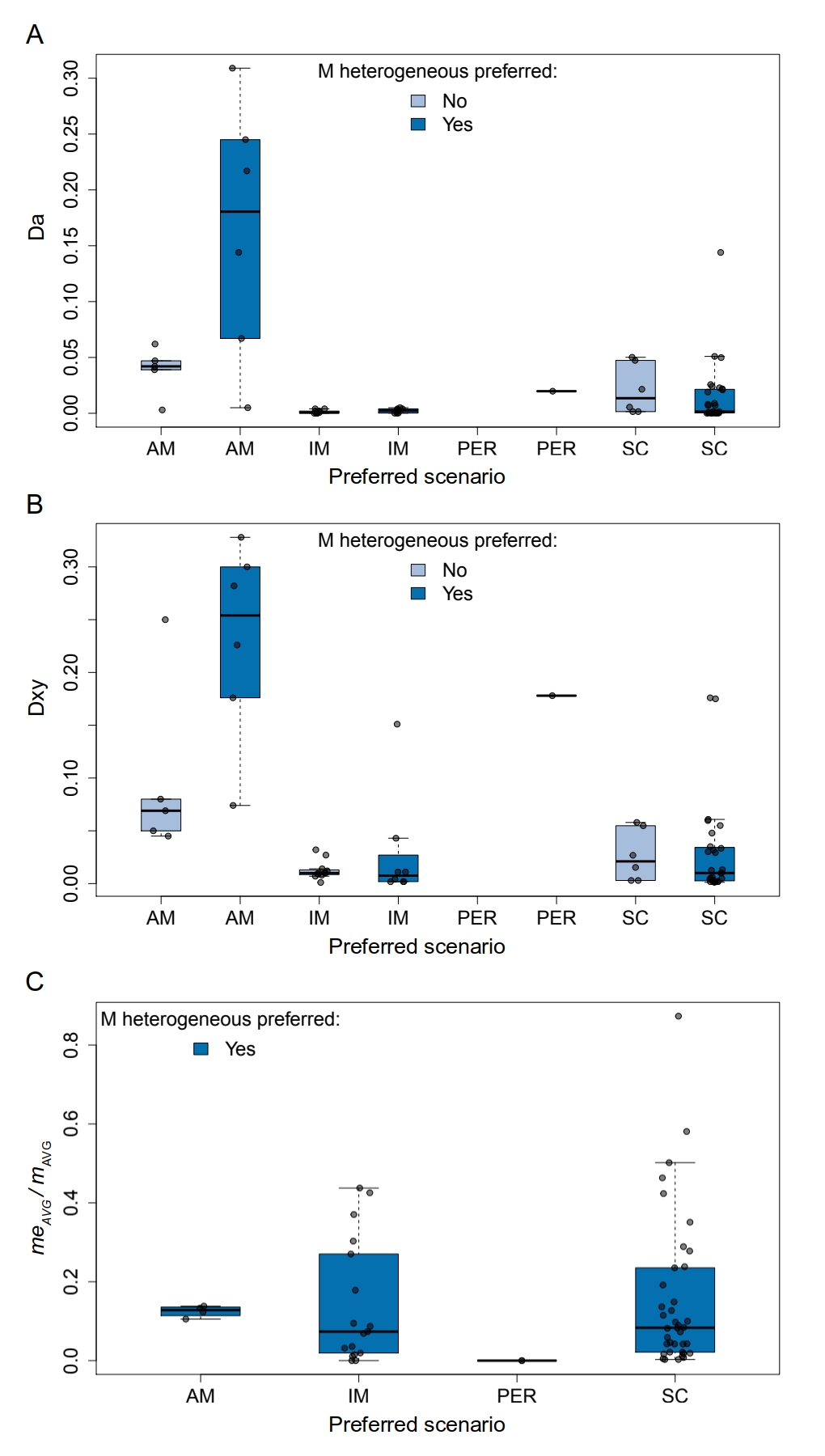


**Supplementary figure 6. Correlation between *F_ST_* and *p*** (n=15)**.** *p* is the fraction of the genome with reduced gene flow. Studies where hetN was not tested were excluded. Other details match Figure 5.


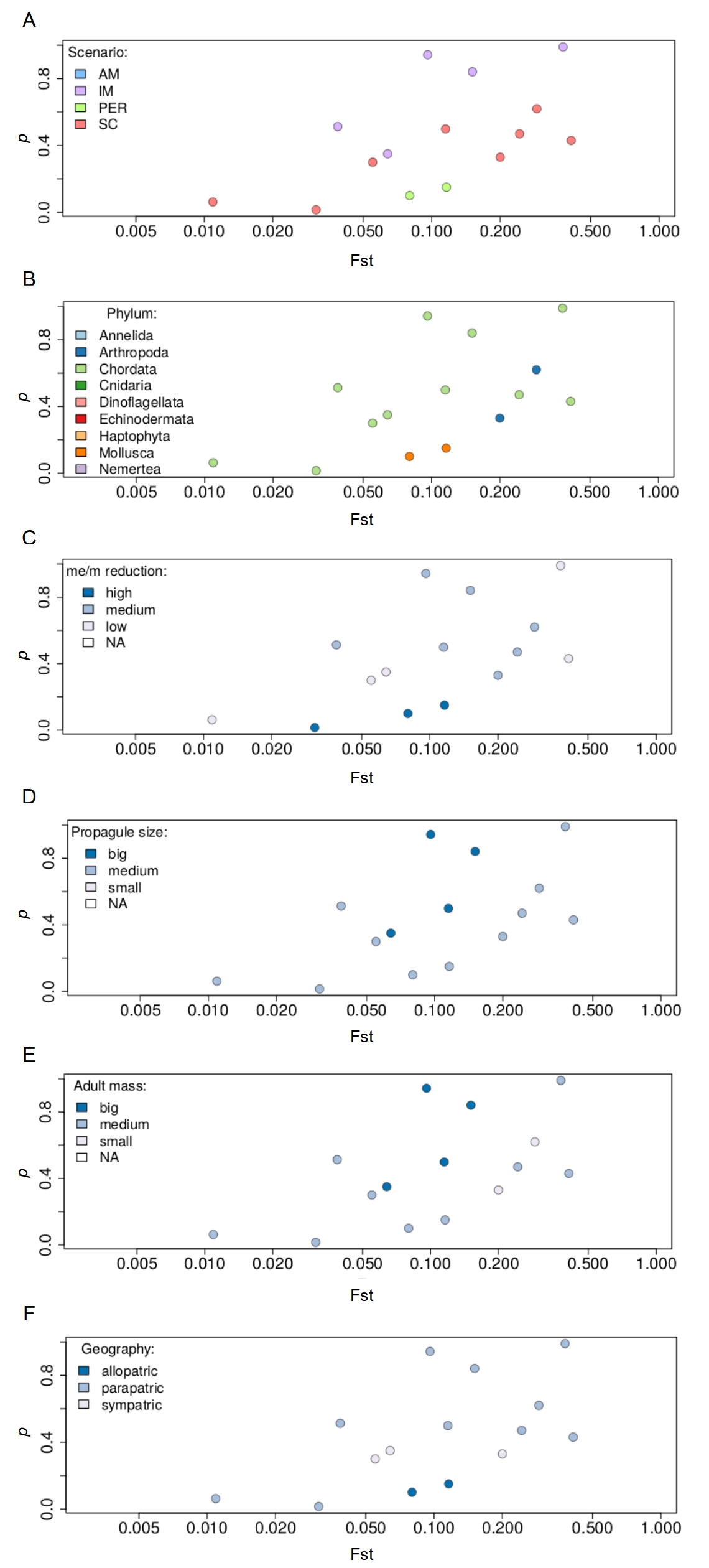


**Supplementary figure 7. Preferred demographic models along the speciation continuum in the sea.** Fraction of the lineage pairs with preferred current gene flow for each divergence category. PER models were excluded. Scenarios were grouped in two categories: i) current gene flow (IM+SC), ii) current isolation (SI+AM), following Roux et al. (2016). Colours correspond to three divergence categories, defined in Roux et al. from *Da* values. A) Dataset we analysed (n=64). B) Dataset we analysed, excluding studies where hetN was not tested (n=56). C) Roux et al. 2016 (n=56).


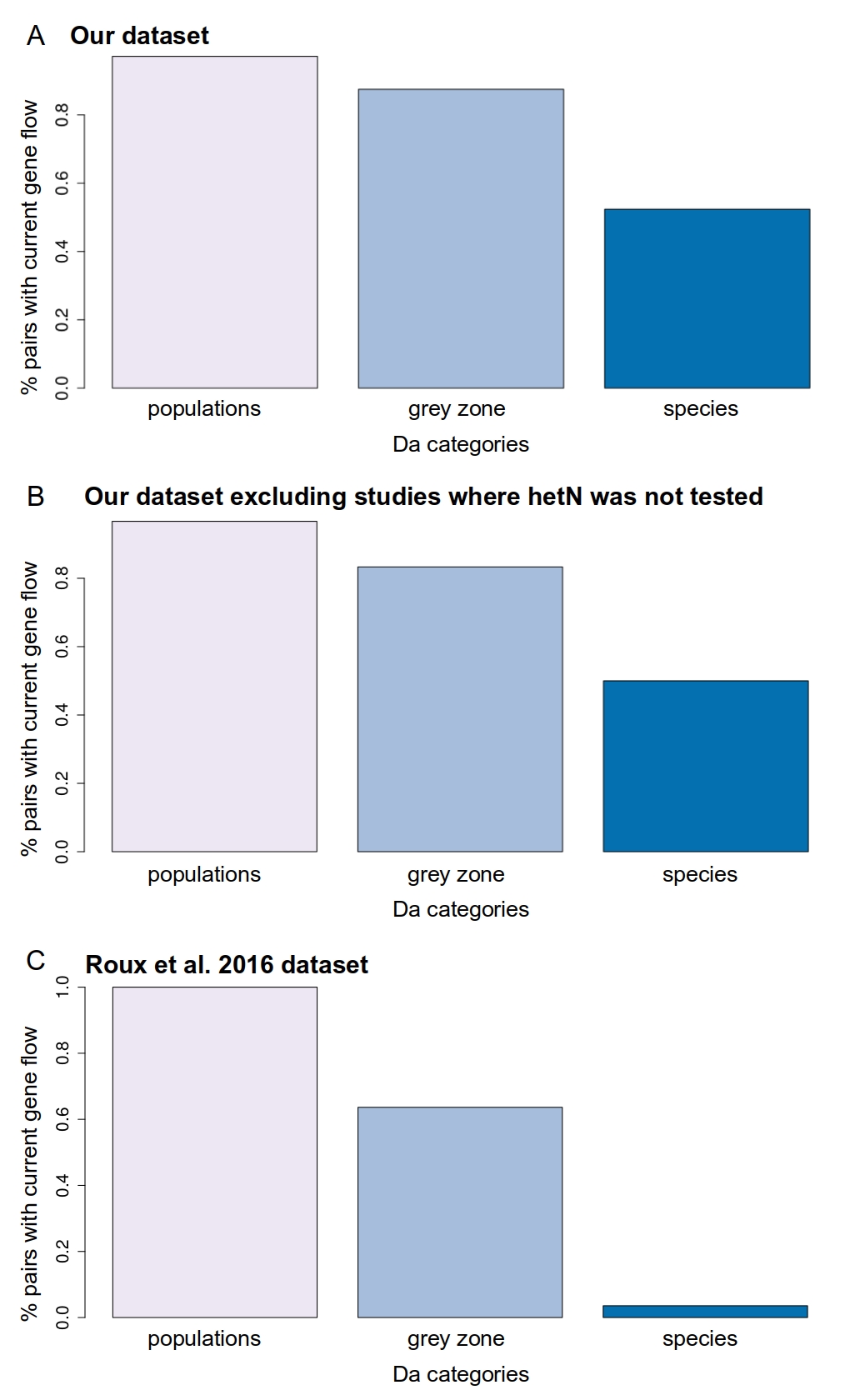


**Supplementary figure 8. Preferred genome-wide heterogeneity of gene flow** **along the speciation continuum in the sea.** Fraction of the lineage pairs with preferred hetM for each divergence category. Only studies where hetM was tested were considered. Colours correspond to three divergence categories defined by *Da* values. A) Dataset we analysed (n=64). B) Dataset we analysed, excluding studies where hetN was not tested (n=55). C) Roux et al. 2016 (n=56).


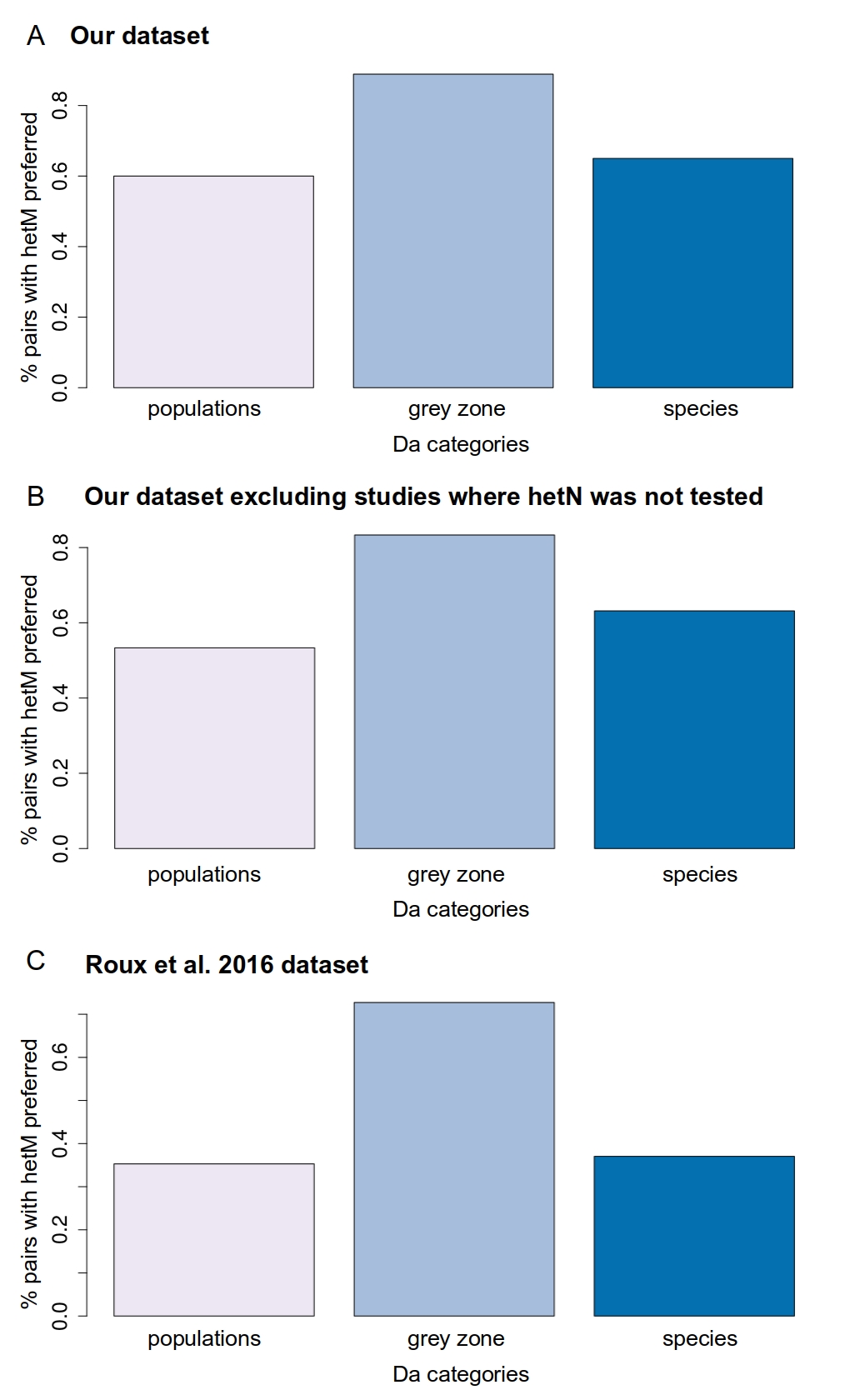


**Supplementary figure 9. Preferred genome-wide heterogeneity of effective size** **along the speciation continuum in the sea.** Fraction of the lineage pairs with preferred hetN for each divergence category. Only studies where hetN was tested were considered. Colours correspond to three divergence categories defined by *Da* values. A) Dataset we analysed (n=65). B) Dataset we analysed, excluding studies where hetN was not tested (n=56). C) Roux et al. 2016 (n=56).


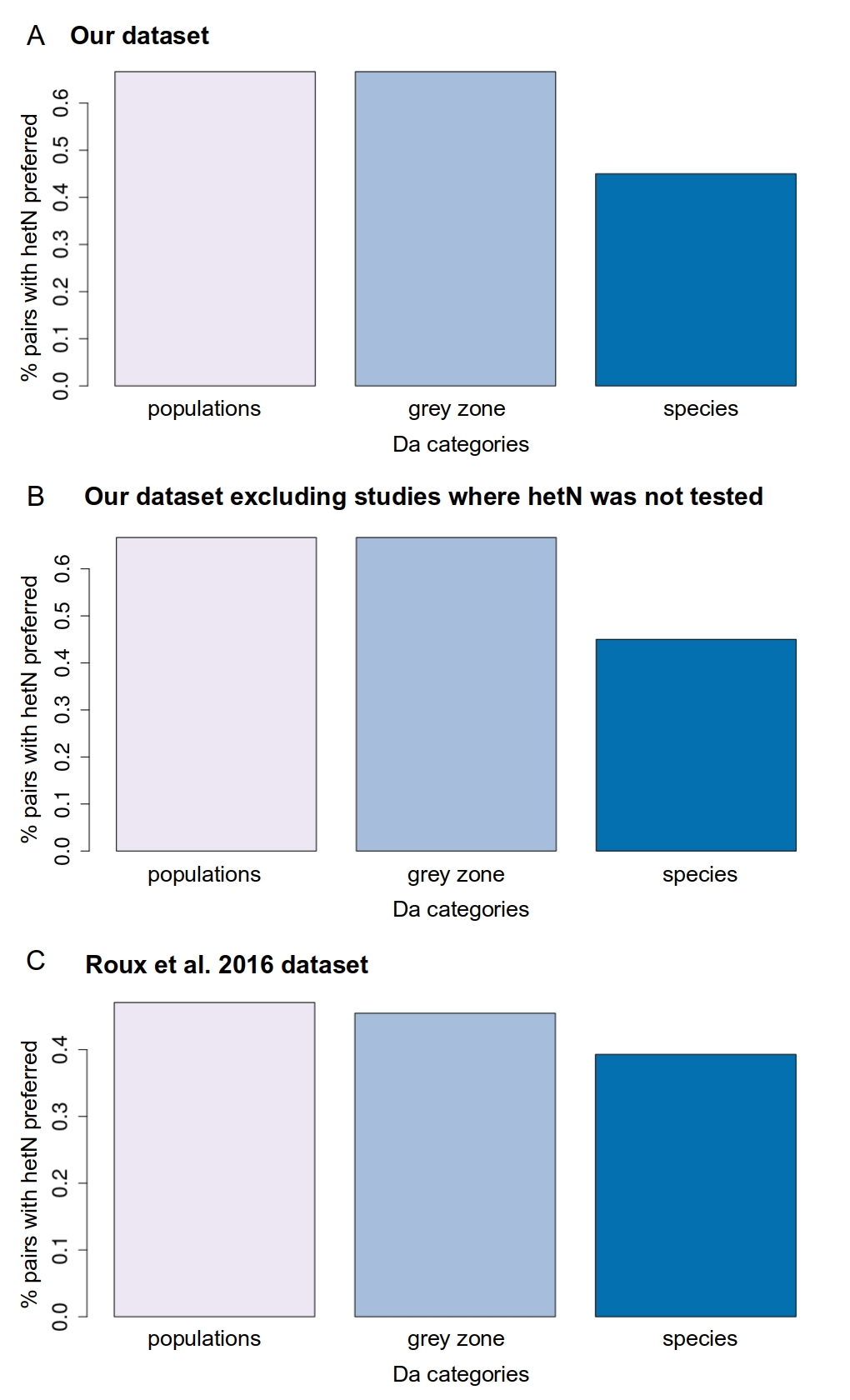


**Supplementary figure 10. Correlation of *m_e_/m* with *F_ST_*** (n=55, **A**) **and with *p*** (n=58, **B**)**.** Colours correspond to the preferred demographic scenarios. In panel (A), the x axis is in log scale.


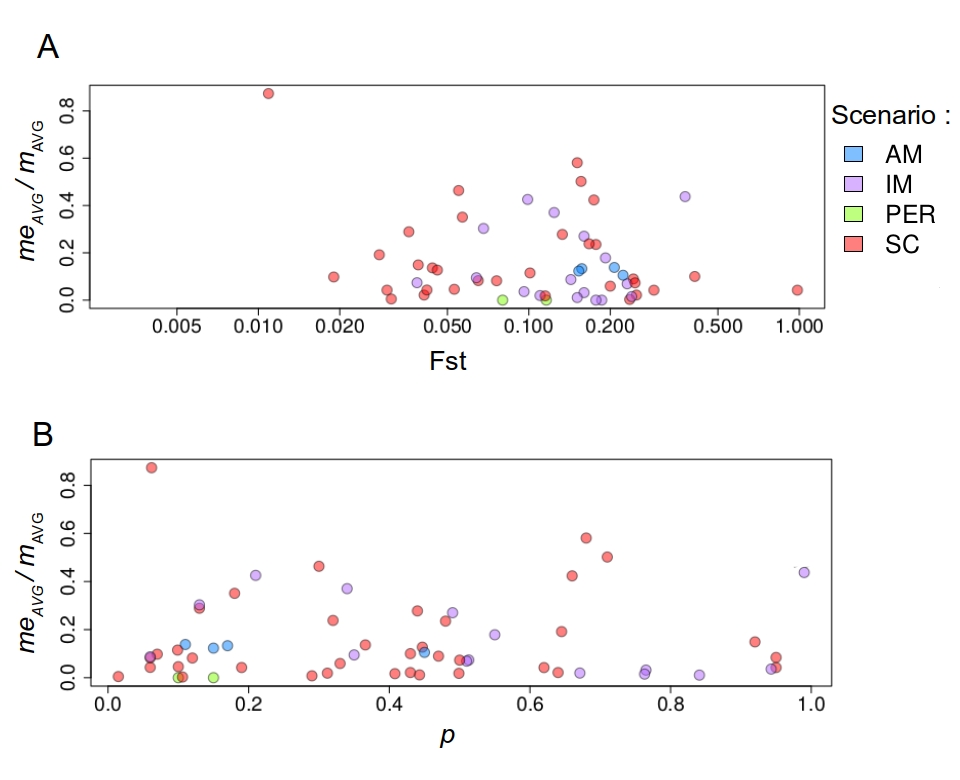

Supplement: Supplementary file 1 — Figures S1‐S10 [file EVA-16-542-s001.docx]
